# K_0.72_Na_1.71_Ca_5.79_Si_6_O_19_ – the first oligosilicate based on [Si_6_O_19_]-hexamers and its stability compared to cyclo­silicates

**DOI:** 10.1107/S2052520624007352

**Published:** 2024-08-30

**Authors:** Volker Kahlenberg, Hannes Krüger, Sonja Garber, Biljana Krüger, Eugen Libowitzky, Stefanie Kröll, Thomas S. Hofer, Josef M. Gallmetzer, Felix R. S. Purtscher

**Affiliations:** a Institute of Mineralogy and Petrography University of Innsbruck Innrain 52 6020Innsbruck Austria; b Department of Mineralogy and Crystallography University of Vienna Josef-Holaubek-Platz 2 A-1090Vienna Austria; c Department of General, Inorganic and Theoretical Chemistry University of Innsbruck Innrain 80-82 A-6020Innsbruck Austria; Academy of Sciences of the Czech Republic, Czechia

**Keywords:** crystal structure, oligosilicate, [Si_6_O_19_] unit, electronegativity equalization method, K_0.72_Na_1.71_Ca_5.79_Si_6_O_19_

## Abstract

Synthesis experiments were conducted in the quaternary system K_2_O–Na_2_O–CaO–SiO_2_, resulting in the formation of a previously unknown compound with the composition K_0.72_Na_1.71_Ca_5.79_Si_6_O_19_. Single crystals of sufficient size and quality were recovered from a starting mixture with a K_2_O:Na_2_O:CaO:SiO_2_ molar ratio of 1.5:0.5:2:3. The mixture was confined in a closed platinum tube and slowly cooled from 1150°C at a rate of 0.1°C min^−1^ to 700°C before being finally quenched in air. The structure has tetragonal symmetry and belongs to space group *P*4_1_22 (No. 91), with *a* = 7.3659 (2), *c* = 32.2318 (18) Å, *V* = 1748.78 (12) Å^3^, and *Z* = 4. The silicate anion consists of highly puckered, unbranched six-membered oligomers with the composition [Si_6_O_19_] and point group symmetry 2 (*C*_2_). Although several thousands of natural and synthetic oxosilicates have been structurally characterized, this compound is the first representative of a *catena*-hexasilicate anion, to the best of our knowledge. Structural investigations were completed using Raman spectroscopy. The spectroscopic data was interpreted and the bands were assigned to certain vibrational species with the support of density functional theory at the HSEsol level of theory. To determine the stability properties of the novel oligosilicate compared to those of the chemically and structurally similar cyclo­silicate combeite, we calculated the electronegativity of the respective structures using the electronegativity equalization method. The results showed that the molecular electronegativity of the cyclo­silicate was significantly higher than that of the oligostructure due to the different connectivities of the oxygen atoms within the molecular units.

## Abstract 2

The first example of a silicate based on [Si_6_O_19_] oligomers is described in detail. The relative stability of [Si_6_O_19_] units compared to cyclic [Si_6_O_18_] rings is discussed using concepts from the electronegativity equalization method.

## 1. Introduction

This paper presents a study on a new phase of the quaternary system K_2_O–Na_2_O–CaO–SiO_2_. Crystalline compounds and subsolidus equilibria in the Na_2_O–CaO–SiO_2_ and K_2_O–CaO–SiO_2_ subsystems have been frequently investigated in the past (Kahlenberg & Hösch, 2002 ▸; Kahlenberg *et al.*, 2010 ▸; Arroyabe & Kahlenberg, 2011 ▸; Schmidmair *et al.*, 2017 ▸; Schmidmair *et al.*, 2018 ▸). However, recent studies have described several new silicates containing calcium as well as sodium or potassium, such as γ-Na_2_Ca_6_Si_4_O_15_ or K_4_CaSi_6_O_15_ (Kahlenberg *et al.*, 2022 ▸; Liu *et al.*, 2021 ▸). This suggests that these systems are not yet as well understood as one might expect. In contrast, the quaternary system containing both alkali oxides remains largely unexplored, and detailed thermo­dynamic or phase analysis data are not yet available.

In silicate glass research, it has been long recognized that the simultaneous presence of both Na^+^ and K^+^ cations in the melt and the resulting glasses can cause significant changes in certain physicochemical properties (Dilmore *et al.*, 1978 ▸; Grund & Jonson, 2009 ▸). This phenomenon known as the *mixed alkali effect* describes how chemical durability, electric conductivity and viscosity can vary in an extremely nonlinear fashion when one alkali oxide is gradually substituted by another (Shelby, 2005 ▸).

To date, only a limited number of potassium–sodium–calcium silicates have been structurally characterized, such as K_1.08_Na_0.92_Ca_6_Si_4_O_15_ (Kahlenberg *et al.*, 2018*a* ▸), Na_1.5_K_0.5_Ca_6_Si_4_O_15_ (Kahlenberg *et al.*, 2018*b* ▸), and NaKCa_4_[Si_9_O_23_] (Kasatkin *et al.*, 2019 ▸). The first two members are mixed-anion silicates and are isostructural with phases of the ternary subsystems. The third compound, corresponding to the mineral patynite, has a previously unknown structure type and belongs to the group of inosilicates based on branched tubular [Si_9_O_23_]^10−^ chains. Additionally, there are indications that combeite (ideally Na_4_Ca_4_Si_6_O_18_) can incorporate small amounts of potassium (Kahlenberg, 2023 ▸). The example of patynite emphasizes the need for a more thorough analysis of the quaternary system Na_2_O–K_2_O–CaO–SiO_2_. It suggests that additional phases may exist, which are not mere solid solutions of end members containing only one alkali cation species. Knowledge of the existence of novel Na–K–Ca silicates is also important for thermodynamic modelling of the system using program packages such as *FactSage* (Bale *et al.*, 2009 ▸). This understanding is crucial for comprehending the processes that occur in slags from biomass combustion (Vassilev *et al.*, 2013 ▸; Santoso *et al.*, 2020 ▸).

In addition to diffraction-based structure investigations, a deeper understanding of crystalline materials can be achieved by computing key physicochemical properties through quantum chemical calculation methods such as density functional theory (DFT) (Argaman & Makov, 2000 ▸; Brink, 2002 ▸; Jones, 2015 ▸; Yu *et al.*, 2016 ▸). This method represents a well established theoretical framework for characterizing chemical systems and has been increasingly successful (Becke, 2014 ▸; Schleder *et al.*, 2019 ▸). However, quantum mechanical approaches have the disadvantage of high computational costs that increase unfavourably with system size. If the research question is based on a basic estimation of crystalline properties, such as electronegativity χ or charge distribution, alternative techniques have been developed that avoid a full quantum mechanical treatment. Electronegativity χ is a particularly suitable parameter that has been used, among others, to characterize and distinguish different silicate-based solid-state systems. This chemical property provides initial information about the charge transfer effects, and thus about the structural stability of materials (Shankar & Parr, 1985 ▸). The *electronegativity equalization method* (EEM) (Mortier *et al.*, 1985 ▸; Mortier *et al.*, 1986 ▸) is a simple parameterized framework that estimates the molecular electronegativity χ_mol_ associated with the partial charge distribution in the system.

Previous investigations of silica structures (including real and hypothetical zeolites with SiO_2_ composition) have shown that the EEM approach can be applied to predict the stability of SiO_2_ polymorphs in a simple and effective way (Van Genechten *et al.*, 1987 ▸; Van Genechten & Mortier, 1988 ▸; Janssens *et al.*, 1995 ▸; Verstraelen *et al.*, 2012 ▸). In this work, an adaptation of the elementary EEM approach has been introduced by using the shielded EEM scheme of the reaction force-field (ReaxFF) method, which allows the extension of this method to more complex silicate systems. The ReaxFF method originates from the molecular dynamic field and has been used for various solid-state characterizations (Senftle *et al.*, 2016 ▸; Leven *et al.*, 2021 ▸). For our specific case, this EEM advancement was positively verified by comparing it with the results of previous investigations on SiO_2_ modifications (Henry, 1997 ▸) and Van Genechten *et al.* (1987 ▸). Thus, the ReaxFF parameters (Joshi *et al.*, 2014 ▸) and our scheme have been shown to provide the same stability trend as proposed in the literature when applied to binary SiO_2_ systems.

This validated EEM approach was used to gain some insight into the stability differences between the exotic hexameric [Si_6_O_19_] oligomer present in the compound under investigation (K_0.72_Na_1.71_Ca_5.79_Si_6_O_19_) and the much more frequent six-membered [Si_6_O_18_] rings existing, for example, in the chemically and structurally related mineral combeite (ideally Na_4_Ca_4_[Si_6_O_18_]; Fischer & Tillmanns, 1987 ▸). In addition, the experimentally determined Raman spectra were interpreted with the aid of a harmonic vibrational analysis (Ozaki *et al.*, 2021 ▸; Wolters & Braun, 2018 ▸) conducted at the HSEsol level of theory (Schimka *et al.*, 2011 ▸).

## 2. Experimental and computational details

### 2.1. Synthesis

For the synthesis experiment, the molar ratio of K_2_O:Na_2_O:CaO:SiO_2_ was 1.5:0.5:2:3. Na_2_CO_3_ (Merck, 99.9%), CaCO_3_ (Merck, >99.9%), K_2_CO_3_ (Alfa Aesar, 99.997%) and SiO_2_ (AlfaAesar, 99.995%) were dried for 24 h at 400°C before being weighed on an analytical balance. Subsequently, a 2 g batch corresponding to the aforementioned molar ratio was thoroughly mixed in a planetary ball mill for 45 min at 600 rpm using ethanol as a grinding fluid. After evaporating the alcohol at 50°C in a hot-air cabinet, the reactants were stored in a desiccator. The high-temperature treatment was carried out in a small platinum capsule with an inner diameter of 5 mm and a length of approximately 35 mm. The lower end of the capsule was closed using a welding apparatus, and 72.8 mg of the reactant mixture was charged into the container. A sintered corundum combustion boat was filled with hollow spheres of alumina, and the capsule was placed vertically inside. The boat was then transferred to a resistant heated chamber furnace and slowly heated from ambient temperature to 700°C. After annealing for 67 h to ensure complete disintegration of the carbonates the sample was removed from the furnace. The observed weight loss corresponded to the expected value for the release of CO_2_. Finally, the upper open end of the capsule was pinched and welded shut. Sealing was performed to prevent evaporation of K_2_O and Na_2_O, which is likely to occur at elevated temperatures. The capsule was then heated from 140°C to 1150°C with a ramp of 5°C min^−1^. After holding the target temperature for 1.5 h, the sample was cooled down to 700°C with a rate of 0.1°C min^−1^ and, finally, quenched in air to ambient conditions by removing the alumina container from the furnace. The closed capsule was weighed before and after the high-temperature treatment. No weight loss was observed, indicating that the container remained intact throughout the synthesis process.

### 2.2. Single-crystal X-ray diffraction

After opening the Pt capsule with a micropincer, the solidified material was transferred to a glass slide and further checked using polarization microscopy. The material was found to consist of transparent, colourless birefringent crystals up to 150 µm in diameter and a glassy isotropic matrix showing conchoidal fracture. The crystals exhibited very good optical quality, displaying a sharp extinction between crossed polarisers. Fifteen crystals were affixed to the tips of glass fibres using nail polish. The crystals were then studied using single-crystal diffraction performed on an Oxford Diffraction Gemini R Ultra diffractometer, which was equipped with a four-circle kappa-goniometer and a Ruby CCD detector. Preliminary diffraction experiments aimed at the determination of the unit-cell parameters and revealed the presence of three different phases: rankinite (Ca_3_Si_2_O_7_), wollastonite (CaSiO_3_), and an unidentified compound with a tetragonal metric that did not match any entries contained in the current version of the Inorganic Crystal Structure Database (Hellenbrandt, 2004 ▸). Hence, the most superior diffraction quality sample of the novel compound was chosen for further structure analysis. A full sphere of reflections up to 26.37 °θ was obtained using Mo *K*α radiation (refer to Table 1 ▸). The data was processed using the *CrysAlis PRO* software package (Rigaku Oxford Diffraction, 2020 ▸). Following indexing, the diffraction pattern was integrated. The data reduction process involved incorporating Lorentz and polarization corrections, as well as applying an analytical numeric absorption correction using a multifaceted crystal model.

The intensity statistics did not provide a clear indication of the presence or absence of a centre of symmetry. Merging the data set in the two potential tetragonal Laue groups 4/*m* and 4/*mmm* resulted in almost identical internal *R* values. Therefore, it was assumed that the diffraction symmetry corresponds to the holosymmetric Laue class. Based on the observed reflection conditions (00*l*): *l* = 4*n*, only the two enantiomorphic space groups *P*4_1_22 and *P*4_3_22 remained. The structure solution was successfully initiated in *P*4_1_22 using direct methods (*SIR2002*; Burla *et al.*, 2003 ▸), which provided a crystal-chemically reasonable starting model. Some missing oxygen atoms were found from a difference Fourier map (*SHEXL97*; Sheldrick, 2015 ▸). The same software was also employed for subsequent full-matrix least-squares refinements. The scattering curves and anomalous dispersion coefficients were obtained from the *International Tables for Crystallography*, Vol. C (Prince, 2004 ▸). Section 3 will provide a detailed analysis of the site populations of the seven non-tetrahedrally coordinated cation sites in the asymmetric unit. The resulting chemical composition from the structure analysis was K_0.72_Na_1.71_Ca_5.79_Si_6_O_19_. The correctness of the absolute configuration was indicated by a value of −0.03 (5) for the Flack parameter (Flack, 1983 ▸). Table 2 ▸ provides the final coordinates, site occupancies, and equivalent isotropic displacement parameters, while Table 3 ▸ lists the anisotropic displacement parameters. Table 4 ▸ summarizes the selected interatomic distances. Structural features were illustrated using *VESTA3* program (Momma & Izumi, 2011 ▸). Bond valence sum calculations have been performed using the parameter sets of Brown & Altermatt (1985 ▸) for Ca–O, K–O and Na–O interactions as well as Brese & O’Keeffe (1991 ▸) for the Si—O bonds (see Table 2 ▸).

### 2.3. Chemical analysis

The crystal that has been used for structural investigations was removed from the glass fibre and studied using wavelength-dispersive X-ray spectroscopy (WDS) analysis using a Jeol Superprobe JXA-iSP100 electron beam microprobe. The nail polish was dissolved with acetone and the crystal was thoroughly cleaned with a drop of water. The crystal was then mounted directly on an aluminium sample holder without any polishing using a small amount of a hot-melt adhesive, and finally coated with carbon. The acceleration voltage was 15 kV and the beam current was 10 nA. The counting times on the peaks were 20 s, with half that time on both sides of the peaks. The average results for three different spots, when normalized to 19 oxygen atoms, are as follows: K_0.81 (5)_Na_1.50 (5)_Ca_5.69 (3)_Si_6.08 (4)_O_19_. The analysis confirms the simultaneous presence of Na, K and Ca, but the resulting stoichiometry differs slightly from that of the structure refinement. The observed differences may be attributed to the fact that the crystal was measured in its natural state, without any polishing. Regrettably, the crystal was lost while attempting to embed it in ep­oxy resin for a second analysis run with a better surface finish. Taking into account the non-ideal conditions of the electron beam microprobe measurements, we consider the agreement between direct WDS and indirect single-crystal X-ray diffraction (SCXRD) chemical analyses to be satisfactory. It should be noted that the silicate glass phase present in the sample has not been analysed. However, it is reasonable to assume that it is enriched in Na_2_O and especially K_2_O in relation to the alkali-free crystalline byproducts observed in the SCXRD screening process.

### 2.4. Raman spectroscopy

**Figure 1:**
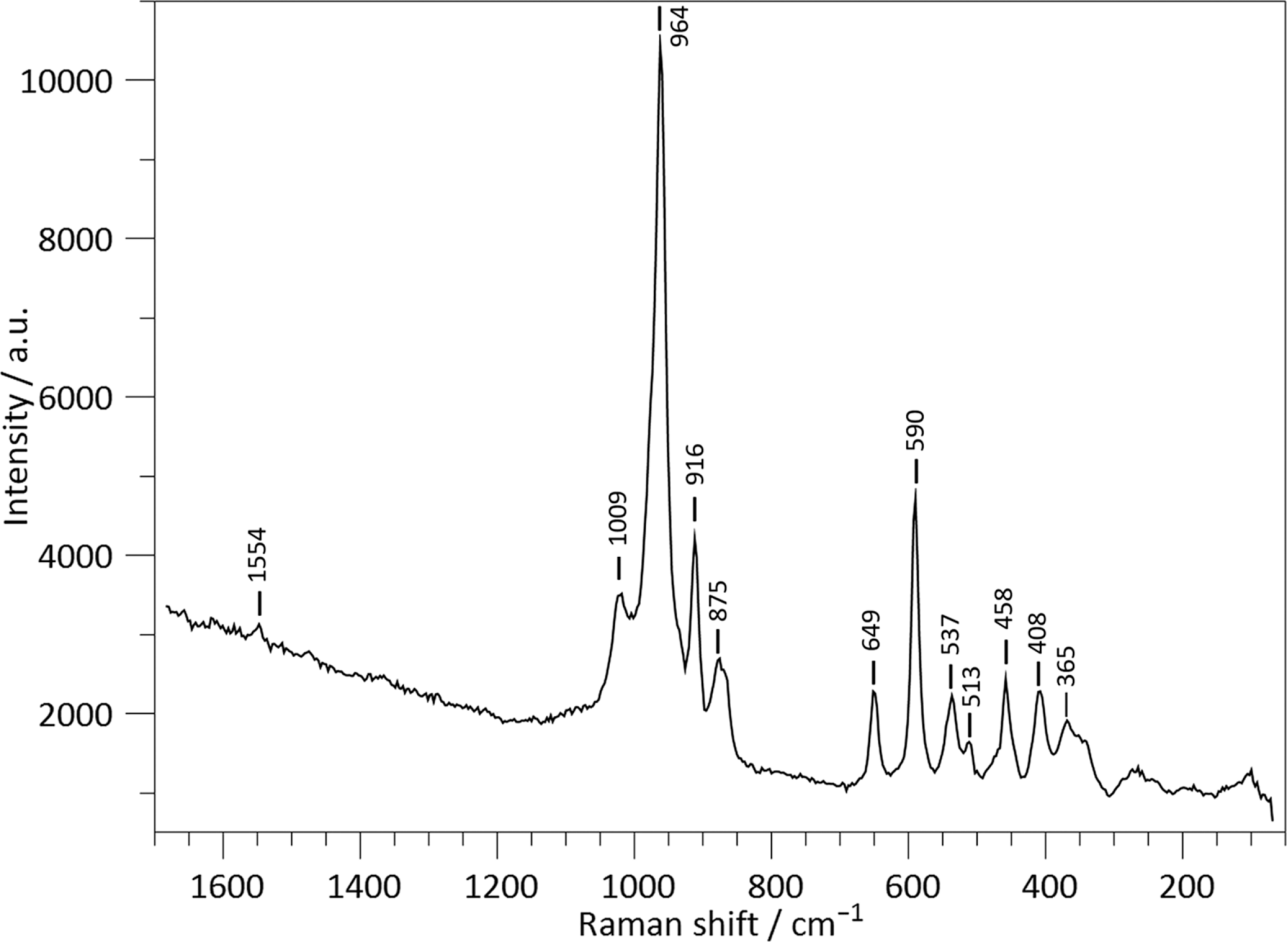
Unpolarized Raman spectrum of a single crystal of K_0.72_Na_1.71_Ca_5.79_Si_6_O_19_.

Raman spectra were collected at the University of Vienna using a Renishaw RM1000 confocal edge filter-based micro-Raman system. The sample analysed was a randomly oriented single crystal of K_0.72_Na_1.71_Ca_5.79_Si_6_O_19_ embedded in nail polish for transport. The sample surface was excited with the blue 488 nm (20 mW) emission line of an Ar^+^ laser using a 50×/0.75 objective lens. The back-scattered radiation (180° configuration) was analysed with a 1200 lines per mm grating monochromator. Unpolarized Raman intensities were collected using a thermo-electrically cooled CCD array detector for 300 s in static grating mode, covering a spectral range of approximately 70 to 1680 cm^−1^. To obtain the final spectrum, faint signals of the nail polish were subtracted. The system has a spectral resolution of 5–6 cm^−1^ and a wavenumber accuracy of 1 cm^−1^, both calibrated with the Rayleigh line and the 521 cm^−1^ line of a Si standard. The confocal setup limited the spatial resolution (both lateral and in depth) to 2−3 µm. Instrument control and data acquisition were performed using the *Grams/32* software (Galactic Ind. Corp.). Fig. 1 ▸ shows the Raman spectrum of the compound under investigation.

### 2.5. Theoretical calculations

In order to investigate the stability of K_0.72_Na_1.71_Ca_5.79_[Si_6_O_19_], the EEM approach is applied by using our in-house developed EEM framework. A detailed theoretical overview of the used EEM approach can be found in Appendix *A*[App appa]. Furthermore, a validation of the approach using a set of test silica structures, is provided in Appendix *B*[App appb]. Section 3.1 will discuss the present oligosilicate K_0.72_Na_1.71_Ca_5.79_[Si_6_O_19_], which contains cation sites with mixed or partial site occupancies. This feature is also observed in the cyclo­silicate combeite, which was chosen as a benchmark to compare the stability of open hexameric oligomers and closed six-membered silicate rings.

Many silicate structures involve cation substitutions on the non-tetrahedral positions. Unfortunately, this cannot be reproduced in a computational structure model. In order to model mixed occupancies, it is necessary to apply an order of magnitude of the supercell size, which is very difficult to achieve or indeed beyond the accessible scope of using DFT for structure optimization.

**Figure 2:**
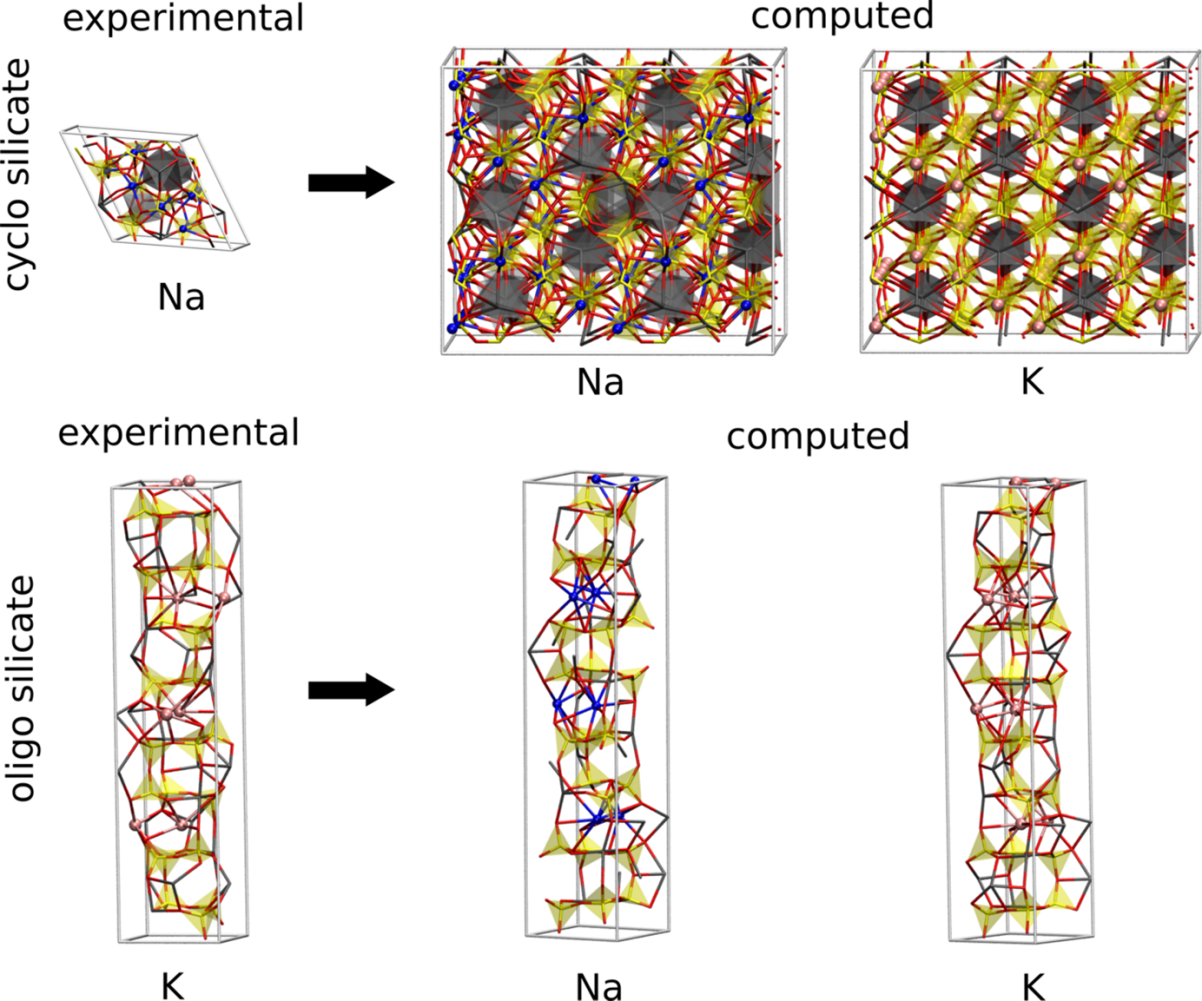
Workflow for generating the model structures for the theoretical calculations. Experimentally determined mixed K–Na occupancies were idealized to either fully populated K or Na sites. Orthogonal supercells were used for the EEM calculations (refer to Table 5 ▸). The labels ‘Na’ and ‘K’ indicate the species considered as the monovalent cation in the corresponding systems. The assignments of the calcium atoms in both the cyclo- and the oligosilicate remained unaltered.

Therefore, reasonably idealized site occupations were employed. Consequently, this work used structural models that considered either full potassium or full sodium occupation, which represent idealized structural models. Regardless of the different occupations of the monovalent ionic sites, the population of the calcium positions remained unchanged. To facilitate the EEM calculation framework, trigonal combeite was transformed into an orthogonal supercell, as illustrated in Fig. 2 ▸. The EEM parameter sets used for the different elements are listed in Table 5 ▸.

Although the calculated structural model differs from the experimentally found K_0.72_Na_1.71_Ca_5.79_[Si_6_O_19_] oligosilicate, the conclusion of the stability study can be considered consistent due to the non-idealized occupancies in the oligosilicate and cyclo­silicate combeite. The objective of the EEM calculations is to investigate the stability of K_0.72_Na_1.71_Ca_5.79_[Si_6_O_19_] oligosilicate in comparison to the cyclo­silicate combeite, with a particular focus on their differing silicate anions. The effects of different crystal structures on their stability have been demonstrated using various SiO_2_ polymorphs as test structures, see Figs. S2 and S3 in the supporting information.

In addition, the study compared the optimized and non-optimized structures to assess the effect of structure optimization, carried out using the same computational settings as in the validation test, see Appendix *B*[App appb]. The initial calculations for the different SiO_2_ test structures described above (see supporting information, Fig. S1), showed that the results obtained using the two different basis sets, pob-DZVP-rev2 and pob-TZVP-rev2 (Vilela Oliveira *et al.*, 2019 ▸), were sufficiently identical (see supporting information, Fig. S2). However, the latter basis set was not used for the calculations of the oligo- and the cyclo­silicate due to its increased computational requirements. To further characterize the idealized oligosilicate, an in-silico vibrational analysis (infrared and Raman) was carried out using the program *Crystal23* (Erba *et al.*, 2023 ▸) at the minimum geometry calculated within the harmonic approximation (Ozaki *et al.*, 2021 ▸). The theoretical line spectra were then subjected to a weighted kernel density estimation using a Gaussian kernel with a width of 2.5 × 10^−3^ cm^−1^ to allow comparison with the experimental spectral data (Wolters & Braun, 2018 ▸). The calculated Raman spectra were then compared to the measured values, taking into account both parallel and perpendicular polarization, as well as the respective total intensity as obtained from *Crystal23*.

## 3. Results

### 3.1. Description of the crystal structure

**Figure 3:**
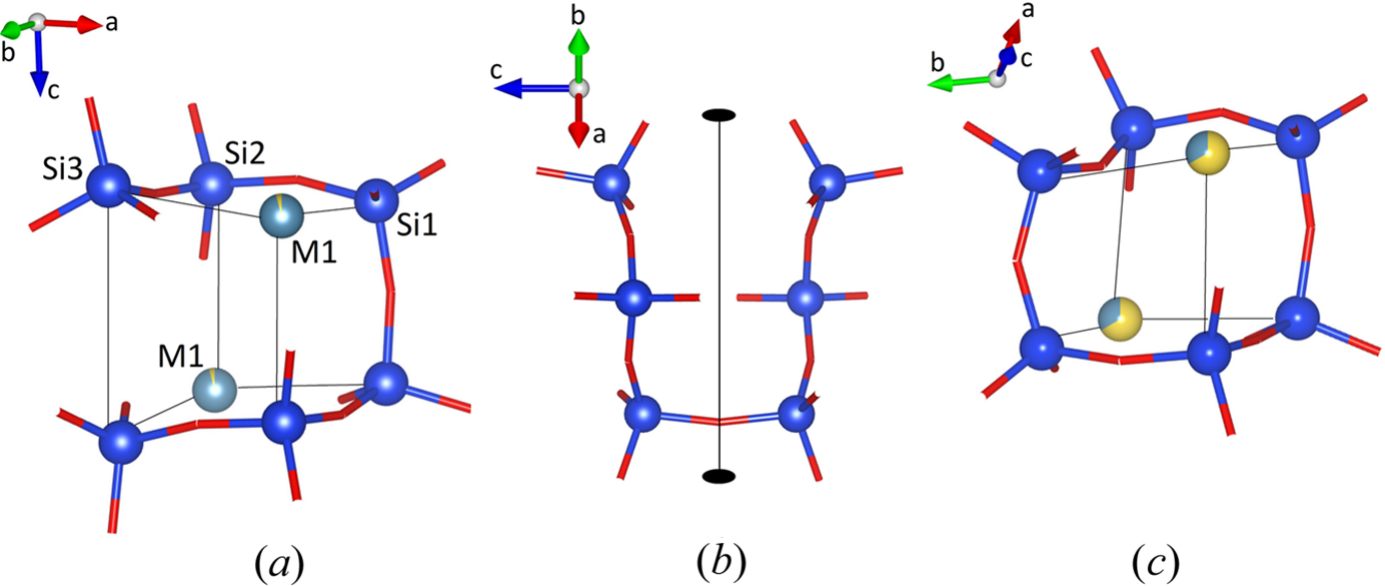
Side views of a single [Si_6_O_19_] oligoanion. (*a*) The six Si atoms occupy the corners of a distorted cube, with the remaining two corners occupied by M1 cations. (*b*) A single [Si_6_O_19_]-cluster has point group symmetry 2 (or *C*_2_). (*c*) The corresponding distorted cube in combeite, ideally Na_4_Ca_4_Si_6_O_18_, contains a cyclic [Si_6_O_18_] ring. The oxygen and silicon atoms are shown in red and blue, respectively. M1 corresponds to a mixed site occupied by calcium (light blue) and sodium (yellow) cations. The sizes of the two-coloured segments refer to the percentages determined from the site-occupancy refinements.

The crystal structure of K_0.72_Na_1.71_Ca_5.79_Si_6_O_19_ is primarily composed of silicate polyanions. These polyanions are formed by the non-cyclic condensation of six [SiO_4_] tetrahedra. According to Liebau’s silicate compendium (Liebau, 1985 ▸), the resulting [Si_6_O_19_] units can be identified as a group of unbranched sixfold tetrahedra. Oligoanions with multiplicities *m* greater than three are uncommon among the numerous natural and synthetic silicate structures that have been determined (Liebau, 1985 ▸). The phase studied in this contribution is the first example of an oligosilicate with *m* = 6. The hexamer present in K_0.72_Na_1.71_Ca_5.79_Si_6_O_19_ is not linear, as shown in Fig. 3 ▸(*a*), but exhibits a high degree of corrugation. The six silicon cations of a single group are located at the corners of an imaginary distorted cube with edge lengths between 3.22 and 4.60 Å. The point group symmetry of a single cluster is 2 (or *C*_2_) [see Fig. 3 ▸(*b*)]. Due to the specific type of connectivity, four tetrahedra exhibit two bridging oxygen atoms (O_br_), while the remaining two [SiO_4_] moieties at the open ends show only one O_br_ each. The individual Si—O bond distances vary considerably, as shown in Table 4 ▸ (ranging from 1.590 to 1.645 Å). However, the obtained values fall within the normal range for oxosilicate structures and their variations follow expected trends. The bond distances between silicon and the bridging oxygen atoms (O8, O9, O10) are consistently longer than the non-bridging Si—O bonds for all three crystallographically independent tetrahedral units. Their deviation from the ideal 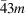 symmetry is also reflected in the Si—O—Si angles, which range from 102 to 119° (refer to Table 6 ▸). The distortion can be quantified numerically using the quadratic elongation (QE) and the angle variance (AV) as defined by Robinson *et al.* (1971 ▸). The corresponding values for the tetrahedra are presented in Table 4 ▸. The conformation of the hexamer can be conveniently expressed using the three torsion angles defined by four successive Si atoms along the group. These values are also listed in Table 6 ▸.

The Na, K and Ca ions within the unit cell must compensate for the 56 negative charges of the silicate anions. The three cation species distribute among seven non-tetrahedrally coordinated positions having six (M1), seven (M2), eight (M3, M4, M5, M7), and ten (M6) next oxygen neighbours. The coordination polyhedron around M1 can be described as a distorted octahedron, whereas the polyhedra around the remaining M positions are more complex. Based on the bond distance analysis, it is concluded that M1–M5 are mixed Na/Ca positions, whereas M7 corresponds to a potassium site. Table 2 ▸ shows the results of an unconstrained refinement of the individual site occupancies for these six positions. As a result, M1–M4 are dominated by Ca, while M5 contains significant amounts of both Ca and Na. The occupancy of the M7 position is only partial. In total, these six sites represent 50.01 (6) charges per cell. Therefore, the remaining M6 position must provide another six positive charges to achieve electroneutrality. It is worth noting that the observed scattering density on the M6 position amounts to almost exactly 18 electrons which is compatible with both K^+^ and Ca^2+^. Occupancy refinements based on laboratory diffraction data are usually unable to directly access mixed site populations containing isoelectronic cations. This is due to the limitation of using only one fixed wavelength, which usually impedes the targeted exploitation of anomalous dispersion effects to enhance the scattering contrast between the two ions. In this particular case, there is only one possible solution to achieve the six missing charges. The M6 site – a special position with a multiplicity of four – must be equally populated with Ca and K, resulting in a 1:1 ratio. Bond valence sum calculations provide additional evidence for the mixed K/Ca occupancy of M6. These calculations allow for an independent, though usually rather rough estimate of the contents of two different atom types sharing the same position (Brown, 2016 ▸). The concentrations obtained using the bond valence parameters of Brown & Altermatt (1985 ▸) for the K–O and Ca–O interactions in combination with the M6—O bond distances given in Table 4 ▸ are as follows: 62% K and 38% Ca. This result is considered to compare well with the percentages determined from charge neutrality requirements. The chemical composition, when normalized for 19 oxygen atoms per formula unit (a.p.f.u.) can be expressed as K_0.72_Na_1.71_Ca_5.79_Si_6_O_19_. This formula compares well with the one obtained by WDS analysis.

**Figure 4:**
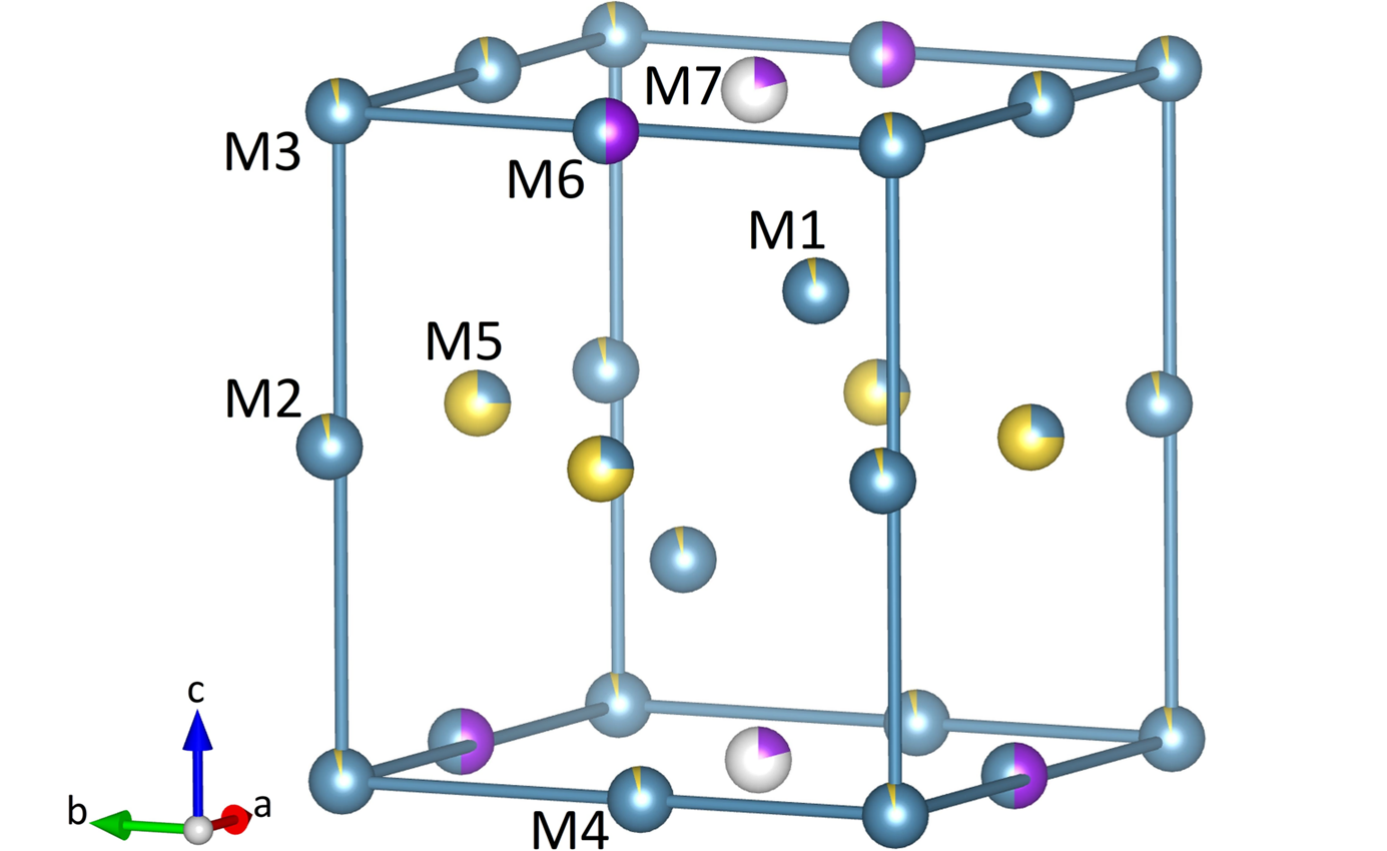
The non-tetrahedral cation positions M2–M7 occupy the corners (M3), the edge centres (M2, M4, M6) as well as the face centres (M5, M7) of pseudocubic modules (tetragonal prisms). The cations are potassium (violet), sodium (yellow) and calcium (light blue). The sizes of the bi-coloured segments indicate the percentages determined from the site-occupancy refinements. Note that M7 is only partially occupied.

**Figure 5:**
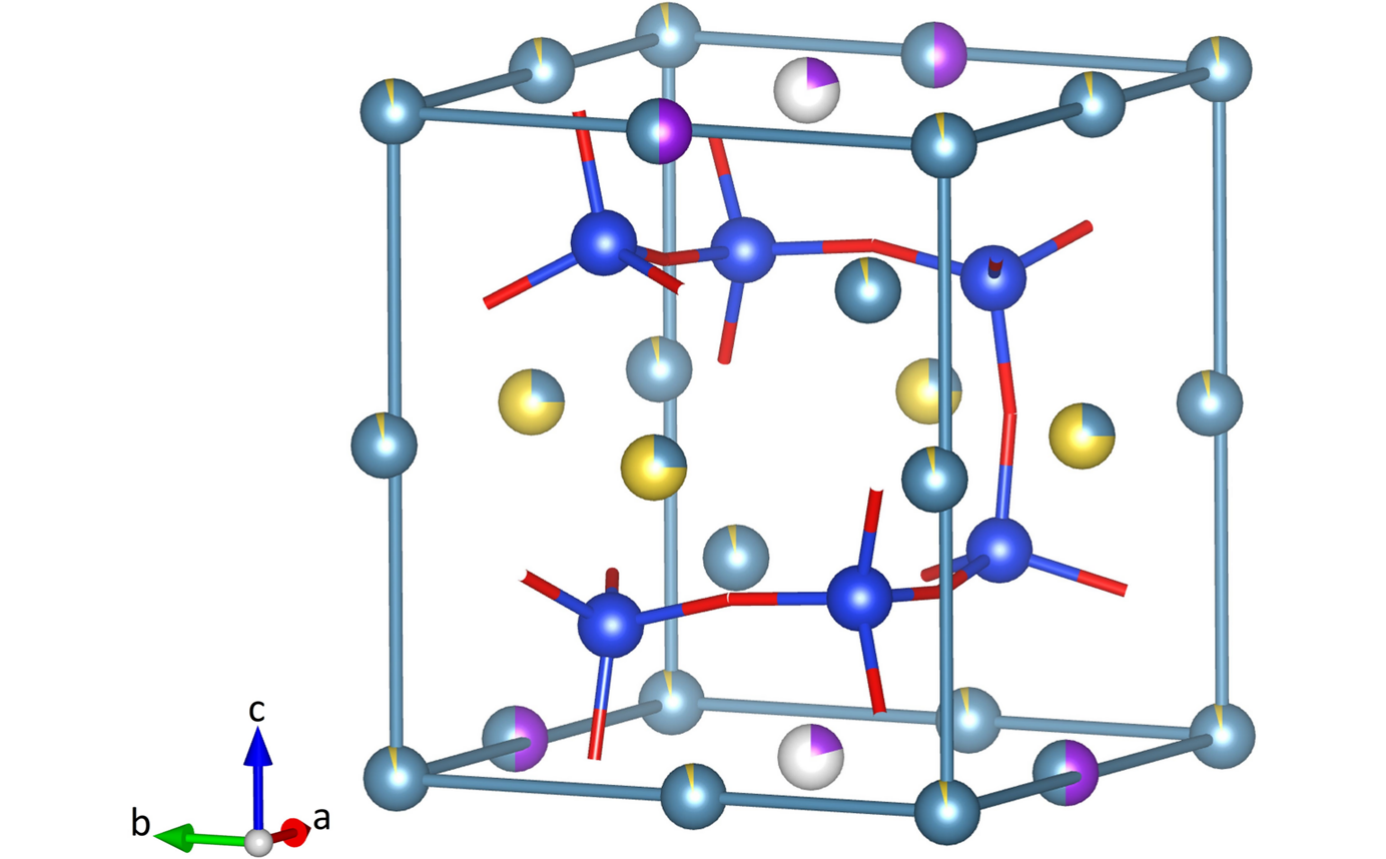
This is a single cube-in-cube arrangement, whereby the smaller distorted cube is defined by the six silicon atoms of the [Si_6_O_19_] unit, in addition to two M1 cations. This cube is situated at the centre of an even larger cube, which is defined by the remaining M positions.

**Figure 6:**
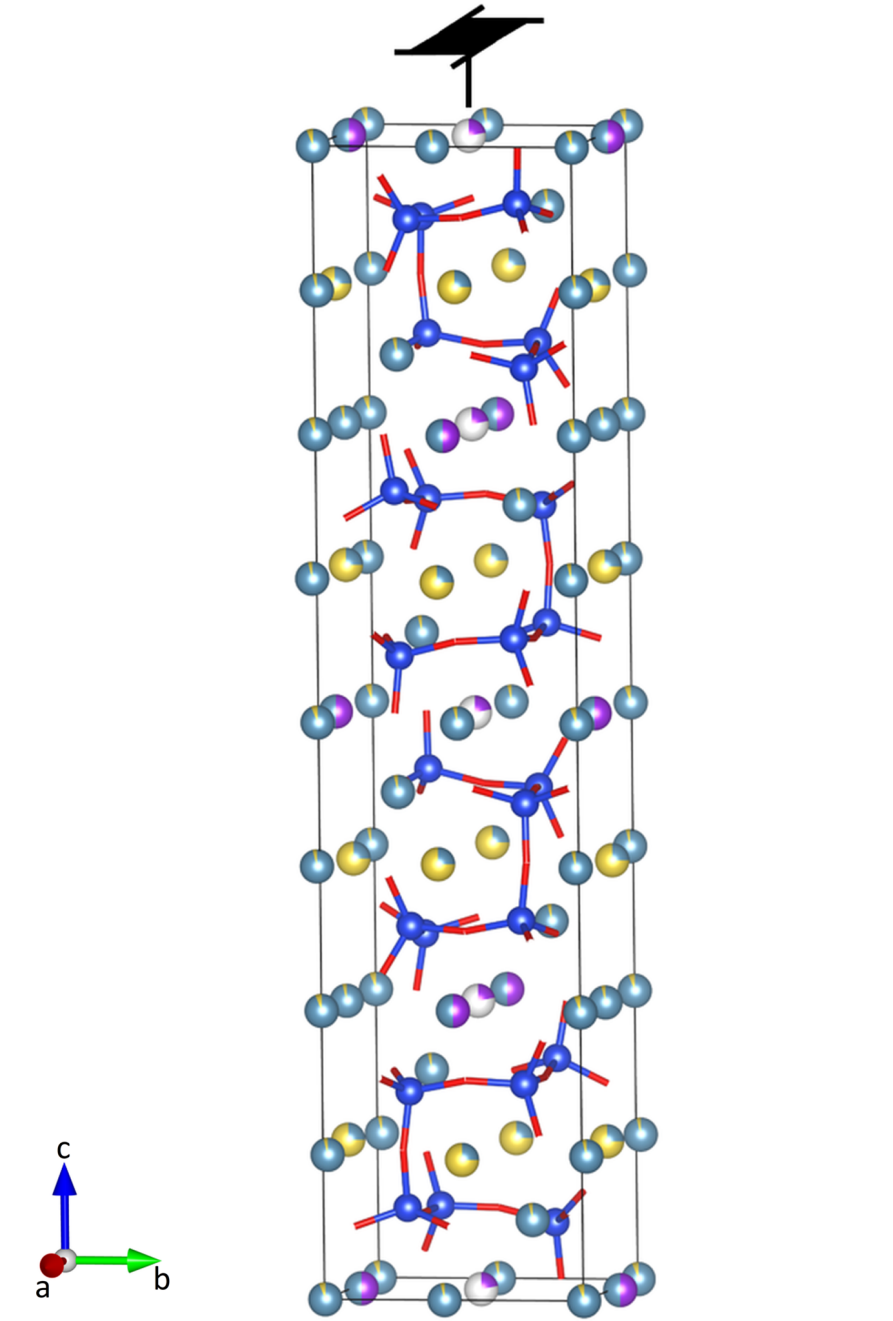
Stacking of four cube-in-cube unit cell defining modules by a 4_1_-screw axis running parallel to [001].

Fig. 4 ▸ shows the arrangement of the substructure of the sites M2–M7. The six positions are located at the corners (M3), as well as the centres of edges (M2, M4, M6) and faces (M5, M7) of pseudocubic modules (tetragonal prisms). The total unit cell contains four of these modules. The dimensions of unit-cell parameters directly reflect the edge lengths of the modules, where *a*′ is equal to *a* and *b*, while *c* is approximately 4 × *a*′. The cation site M1, which is octahedrally coordinated, is located at the remaining two opposite corners of the smaller cubes defined by the six Si atoms belonging to a single hexamer [refer to Fig. 3 ▸(*a*)]. These smaller cubes occupy the barycenters of the larger pseudocubic modules (refer to Fig. 5 ▸). The entire unit cell contains four of the cube-in-cube modules that are stacked by applying the 4_1_-screw axis running parallel to [001] (refer to Fig. 6 ▸).

### 3.2. Topological aspects

**Figure 7:**
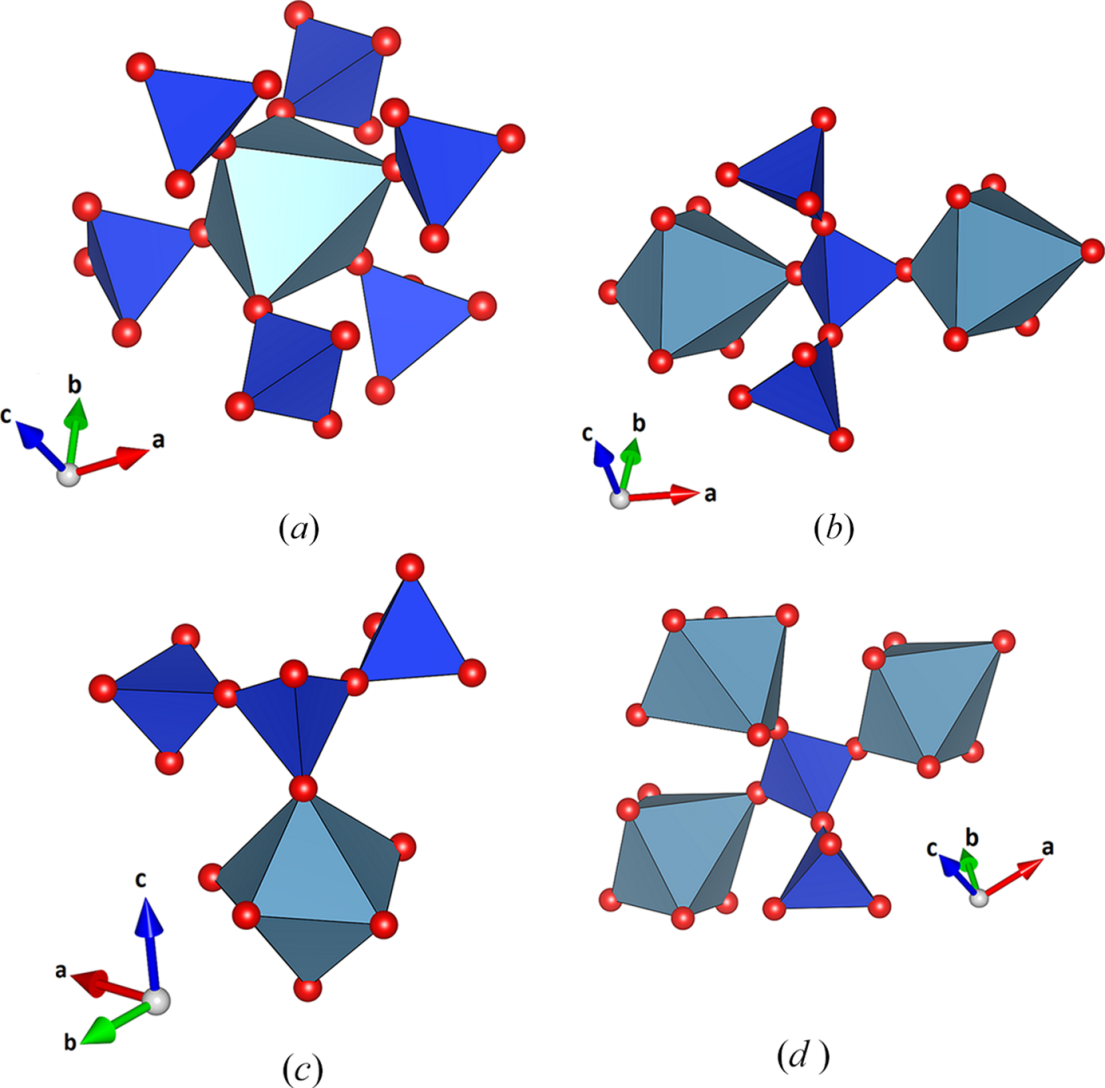
Polyhedral micro-ensembles of the crystallographically independent octahedral (M1) and tetrahedral (Si) nodes: (*a*) {6,6,18} (for M1), (*b*) {4,4,16} (for Si1), (*c*) {4,3,11} (for Si2) and (*d*) {4,4,18} (for Si3).

The crystal structure can be understood differently as a mixed tetrahedral–octahedral framework based on [SiO_4_] and [M1O_6_] units, which contains voids of various sizes that host the remaining Na, Ca and K ions. The mixed framework has been topologically characterized in detail, including coordination sequences and extended point symbols, using the program *ToposPro* (Blatov *et al.*, 2014 ▸). Therefore, the framework is represented by a graph consisting of vertices (T sites containing Si1, Si2 and Si3, M1 site as well as O atoms) and edges (bonds) connecting them. The nodes of the graph can be classified based on their coordination sequences {*N_k_*} (Blatov, 2012 ▸), which is a set of integers {*N_k_*} (*k* = 1,..,*n*), representing the number of sites in the *k*-th coordination sphere of the T/M or O atom selected as the central one. Table 7 ▸ summarizes the corresponding values for the symmetrically independent T sites and the M1 position up to *n* = 12. Additionally, the extended point symbols (Blatov *et al.*, 2010 ▸) that list all shortest circuits for each angle for any non-equivalent framework atom have been determined and are also provided in Table 7 ▸. Finally, the polyhedral micro-ensembles or PMEs have been constructed (see Fig. 7 ▸). On the lowest sublevel they are formed for each octahedron and tetrahedron in the asymmetric unit by considering all directly bonded [M1O_6_] and [SiO_4_] groups. They represent a geometric interpretation of the coordination sequences up to the index *k* = 3. The PMEs of the first sublevel observed for the M1 nodes can be described as follows: each [M1O_6_] octahedron is immediately linked to six tetrahedra. The M1 PME can be denoted as {6,6,18} using the classification based on the calculation of the coordination sequences up to *k* = 3 (Ilyushin & Blatov, 2002 ▸). The PMEs of the three crystallographically independent tetrahedral Si nodes conform to {4,4,16} (for Si1), {4,3,11} (for Si2), and {4,4,18} (for Si3) [refer to Figs. 7 ▸(*a*) to 7 ▸(*d*)].

### 3.3. Raman spectroscopy

**Figure 8:**
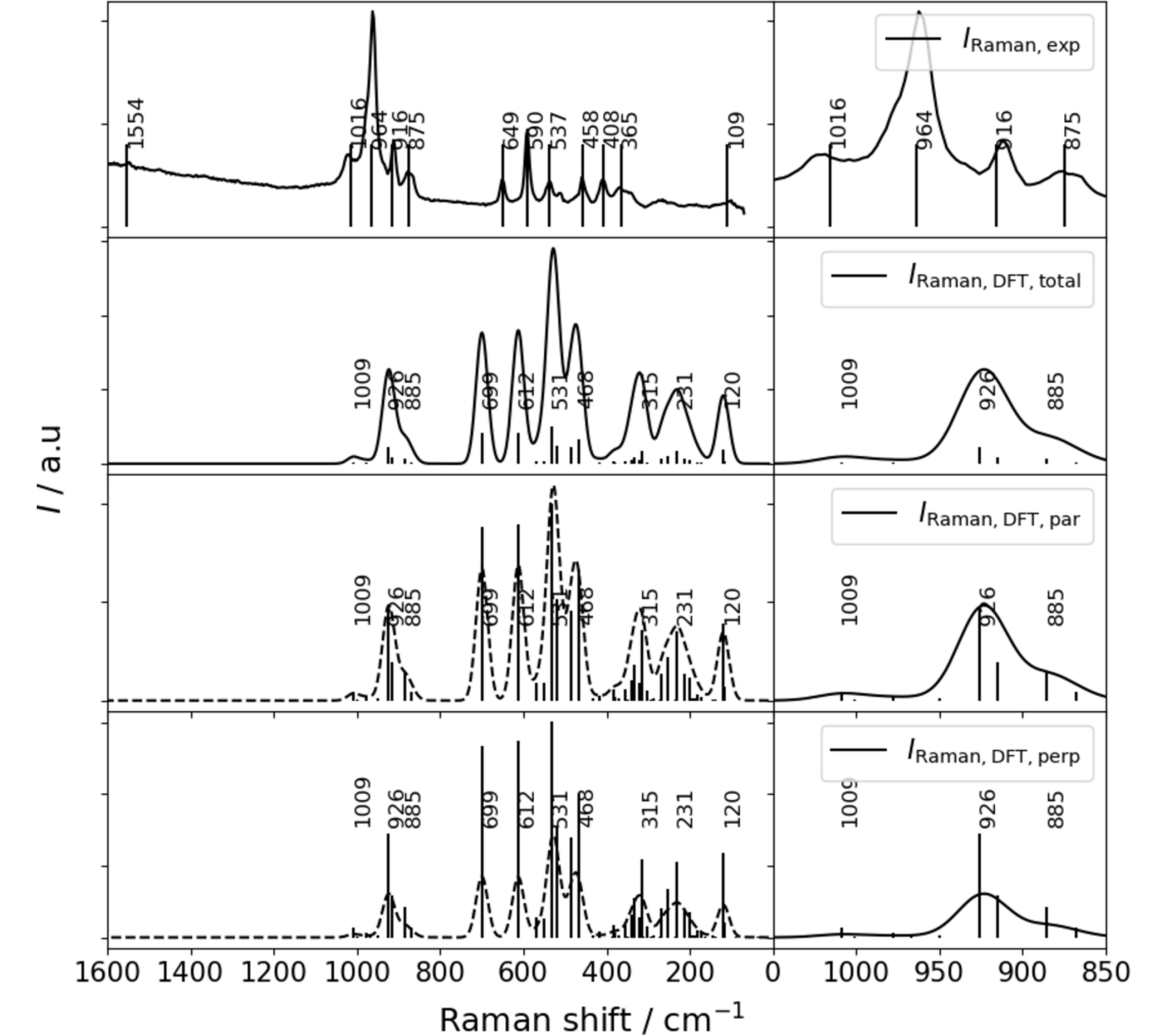
Comparison of the measured and calculated Raman spectra of the oligosilicate is presented. The calculated spectra are separated into contributions associated with parallel and perpendicular polarization as well as for the total intensity, as obtained from the output of the program *Crystal23*. The left-hand side shows the complete spectra, while the right-hand side presents a close-up of the main silicate stretching bands.

As the present compound is the first example of a silicate based on [Si_6_O_19_] units, there are no other spectroscopic data available for crystalline materials based on this moiety in the literature. However, Raman spectra for chemically related oligosilicates with [Si_3_O_10_] units, such as K_2_Ca_3_Si_3_O_10_ (Arroyabe *et al.*, 2011 ▸), have been reported. The band assignments of these authors can be helpful for interpreting the basic features of the spectrum of K_0.72_Na_1.71_Ca_5.79_Si_6_O_19_. Fig. 1 ▸ shows the experimental Raman spectrum, which displays at least 12 bands. The modes above about 800 cm^−1^ are typically attributed to Si—O stretching vibrations of the [SiO_4_] tetrahedra within the larger oligomers, while those in the range between 350 cm^−1^ and 800 cm^−1^ are assigned to O—Si—O and Si—O—Si bending vibrations. The modes within the low-frequency range (< 350 cm^−1^) correspond to (Na,K,Ca)–O vibrations. Fig. 8 ▸ presents a comparison between the calculated Raman spectra and their experimental counterparts. Table S3 summarizes the numerical data. Comparison between the perpendicular and parallel contributions of the total Raman spectral patterns exhibits the same bands with effectively the same intensities. The main experimental bands are located in the region between 1016 cm^−1^ and 875 cm^−1^. The calculated bands exhibit minor deviations of a few wavenumbers, while the intensities are significantly lower than the experimental data. The experimental results display several sidebands with much lower intensity in the 649 cm^−1^ to 109 cm^−1^ region. Despite the reduced intensity, the calculated bands are in good agreement with the experimental reference. The observed differences can be attributed to four factors. First, the non-tetrahedral cation sites are only partially occupied, which cannot be reflected in the harmonic frequency calculation. Second, the use of an energy-minimized structure inherently involves a 0 K treatment, which only allows for a relative measure of stability. Third, experimental Raman intensities are strongly contingent upon the laser wavelength, the sensitivity curve of the used CCD detector, the transmittance of the grating, and other optical components of the system. Finally, the crystal was not of gem quality and may have contained inclusions of the other two phases that crystallized concurrently.

### 3.4. EEM calculations

In addition to calculating harmonic frequencies, we estimated molecular electronegativities using the EEM method. In Fig. S3, EEM validation results based on various silica structures can be found. The results show that the adjusted EEM approach can accurately replicate the sequence of the molecular electronegativity values χ_mol_ of chemically simple SiO_2_ polymorphs, such as α-cristobalite, α-quartz, coesite and stishovite, as previously reported by Henry (1997 ▸) and Van Genechten *et al.* (1987 ▸), respectively. In this study, it was observed that electronegativity increases with increasing unit-cell volume and it converges towards a constant value as expected from the EEM approach (refer to Fig. S2). The optimal supercell size for achieving balance between accuracy and computational costs is approximately 40 Å. Furthermore, it is important to consider the supercell size as isotropic as possible to avoid any artefacts caused by anisotropic orientation. This will ensure a spatially consistent treatment of the radially symmetric Coulomb interactions that contribute to the potential considered in the EEM approach [refer to equation (5), Appendix *A*[App appa]]. Table 8 ▸ provides the optimal trade-off between an isotropic supercell and an appropriate unit-cell size to reach at least the plateau area of the molecular electronegativity χ_mol_ for both the oligo- and the cyclo­silicate. It summarizes the parameters of the orthogonal geometry-optimized supercells that were constructed using the primitive unit cells obtained via energy minimization of the structure, including the lattice parameters, at the HSESol / pob-DZVP-rev2 level of theory. As the pob-TZVP-rev2 and pob-DZVP-rev2 bases effectively yielded the same structural parameters, the latter was deemed sufficient for this work.

**Figure 9:**
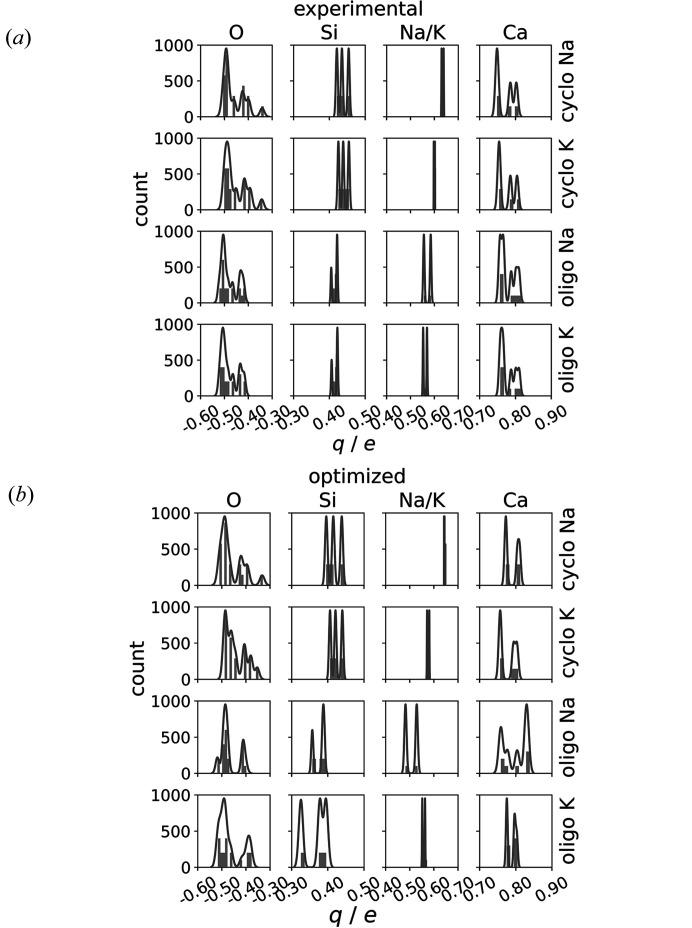
Comparison of the element-wise charge distributions based on the experimental (*a*) and optimized (*b*) structures, as well as the different elemental occupations of Na and K in the oligo- and the cyclo­silicate. The distribution is represented by histograms obtained through kernel density estimation.

Fig. 9 ▸ displays the charge distributions of the experimental (*a*) and optimized (*b*) structures resulting from the EEM calculations. The experimental structures exhibit similar results with respect to their geometry optimized counterparts. The comparison shows that the charge patterns are in good agreement with each other, with only minor shifts in the individual charge distributions. However, the charge distribution shape is broader in the experimental geometry than in the respective optimized structure. On the other hand, the charge patterns for the experimental cyclo­silicate with Na and K occupation are very similar, with only a small shift being visible in the Na and K charge distribution. The oligosilicate exhibits a similar behaviour to the cyclo­silicate. However, the Na charge distribution of the oligostructure is slightly broader than that of the K case, as observed in the results obtained using the optimized crystal structure. The Ca charge distribution is very similar for all investigated models. The main difference between the cyclo- and oligosilicate lies in the patterns of the O and Si charge distributions. The Si charge distribution in the cyclo­silicate model displays three distinct peaks in the range of 0.4 e to 0.5 e. In contrast, the oligostructure only shows two peaks, which are slightly shifted to lower values, and the peak near 0.35 e is absent. The charge distribution of the O atoms follows a similar trend. The cyclo­silicate exhibits several individual peaks in the range of −0.6 to −0.3 e. For the oligosilicate, the peak near −0.35 e is not present, and the remaining contributions are again shifted to smaller values. These differences are evident in both the experimental and the geometry-optimized structures.

**Figure 10:**
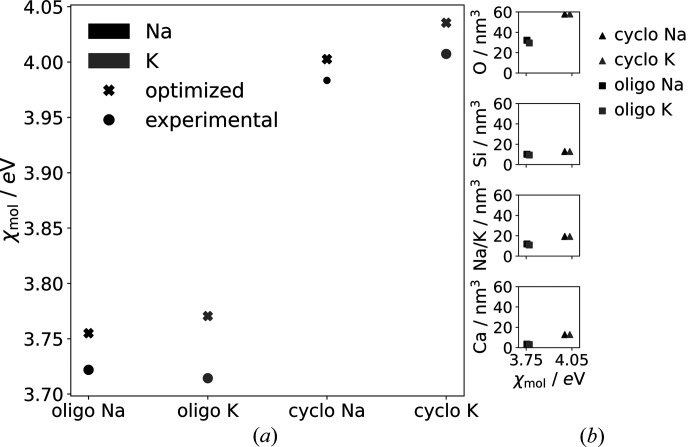
(*a*) Comparison of the molecular electronegativities between the oligosilicate and the cyclo­silicate with full Na and K occupancies using optimized and non-optimized idealized experimental structures. (*b*) Element density (numbers of elements per nm^3^) versus molecular electronegativity between the geometry-optimized cyclo- and oligosilicate with Na/K occupation.

Fig. 10 ▸(*a*) displays the associated molecular electronegativities of the experimental and geometry-optimized structures for the cyclo- and oligosilicate, respectively, considering full Na and K occupation, respectively. The electronegativity values reveal a significant difference between the oligo- and the cyclo­silicate, indicating the latter as the more stable structure (see also the results for the silica polymorphs shown in Fig. S3). Furthermore, there are small differences between the geometry of the optimized and experimental structures, which are more pronounced for the oligosilicate. Additionally, the K occupation for both silicate types leads to slightly higher values, indicating improved stability properties.

Fig. 10 ▸(*b*) illustrates the element density versus the molecular electronegativity plot, highlighting the varying impact of the ions on the molecular electronegativities. The data indicates that the difference in the oxygen density O per nm^3^ between the cyclo- and the oligosilicate is around 30 elements per nm^3^, while the other elements show considerably smaller differences of at most 10 elements per nm^3^. This suggests that the variation in molecular electronegativity can be largely explained by differences in charge transfer involving the oxygen atoms. Only slight differences in element densities are observed between the Na- and K-occupied models for the cyclo­silicate. These differences can be directly correlated with the different cell volumes of the optimized structures.

## 4. Discussion

Unbranched oligoanions [Si*_m_*O_3*m*+1_]^−(2*m*+2)^ based on the condensation of a finite number *m* of [SiO_4_] tetrahedra are rare among natural and synthetic silicates. Although structures containing [Si_2_O_7_] groups (*m* = 2) are still common, the number of representatives for the cases with *m* > 2 appears to decrease with increasing *m* (Liebau, 1985 ▸). Wierzbicka-Wieczorek *et al.* (2010*a* ▸,*b* ▸) provide a comparatively recent summary of oligosilicates with *m* = 3 and *m* = 4. Only one compound each has been identified so far for *m* = 5, 8, 9 and 10: Na_4_Sn_2_[Si_5_O_16_]·H_2_O (Safronov *et al.*, 1983 ▸); Mg_15.61_Sc_1.37_(Mg_0.30_Si_0.02_)[Si_8_O_25_]_2_; Mg_17.40_Sc_1.49_(Mg_0.15_Si_0.11_)[Si_9_O_28_]_2_; Mg_19.60_Sc_1.28_(Mg_0.04_Si_0.22_)[Si_10_O_31_]_2_ (Takéuchi *et al.*, 1984 ▸). Pb_7_[Si_6_O_19_] was found to be composed of heptameric [Si_7_O_22_] and tetrameric [Si_4_O_13_] units in a 2:1 ratio (Siidra *et al.*, 2014 ▸).

Medaite (Mn^2+^_6_[V^5+^Si_5_O_18_(OH)]; Gramaccioli *et al.*, 1981 ▸) is also of interest due to the [VSi_5_O_18_(OH)]^12−^ oligoanion, which contains five [SiO_4_] or [SiO_3_(OH)] groups and an additional terminal [VO_4_] tetrahedron. Depending on the strictness of the silicate classification, medaite could be assigned either to the class with *m* = 6 (if tetrahedrally coordinated cations other than Si are considered) or to the class with *m* = 5 (if only SiO_4_ units are counted).

To the best of our knowledge, the present compound represents the first silicate in which [Si_6_O_19_] anions have been identified. There are no apparent structural similarities between medaite and the K–Na–Ca silicate described in this paper. Furthermore, the conformation of the oligoanions is quite different. In contrast to the highly corrugated arrangement of the hexamers in K_0.72_Na_1.71_Ca_5.79_Si_6_O_19_, the six tetrahedra in the corresponding cluster observed in medaite show an almost linear sequence. As phosphates and silicates frequently share similar features in their crystal chemistry (Averbuch-Pouchot & Durif, 1996 ▸), it was obvious to search also for crystalline compounds based on [P_6_O_19_] clusters. Only one representative has been reported so far: Ca_4_[P_6_O_19_] (Höppe, 2005 ▸). The [P_6_O_19_] groups have a strongly folded geometry, but the three different P—P—P—P torsion angles within the group are either almost 180° or 0°. This means that the folding of the hexamer is restricted to the plane defined by the six phospho­rus atoms and does not have any out-of-plane component.

A few aluminates and gallates containing folded [Al_6_O_19_] and [Ga_6_O_19_] units that are more closely related to the present silicate have been reported in the literature. These include Sr_10_Al_6_O_19_ (Kahlenberg, 2002*a* ▸), isostructural α-Sr_10_Ga_6_O_19_ (Kahlenberg, 2001 ▸; Krüger *et al.*, 2009 ▸) and β-Sr_10_Ga_6_O_19_ (Kahlenberg, 2002*b* ▸). In all three cases, the three torsion angles T—T—T—T (T: Si, Al and Ga) are approximately +90° (‘+’) or −90° (‘−’). The conformation of the hexamer in K_0.72_Na_1.71_Ca_5.79_Si_6_O_19_ is (+, −, +) and also occurs in the β-Sr_10_Ga_6_O_19_. However, due to the centrosymmetry of the space group of the oxogallate, 50% of the [Ga_6_O_19_] groups display the inverted conformation (−, +, −). In centrosymmetric Sr_10_Al_6_O_19_ and α-Sr_10_Ga_6_O_19_ different pairs of conformations are realized: (+, −, −) and (−, +, +), respectively.

The similarity between K_0.72_Na_1.71_Ca_5.79_Si_6_O_19_ and β-Sr_10_Ga_6_O_19_ extends beyond the conformation of the oligomers. Similar to the silicate phase, the [Ga_6_O_19_] units also centre pseudo-cubic modules defined by the non-tetrahedrally coordinated cations (Kahlenberg, 2002*b* ▸). There is a slight difference between the distorted cube in β-Sr_10_Ga_6_O_19_ and the oligosilicate. This difference is due to additional strontium ions occupying the very centre of each distorted cube in the β-phase, whereas the corresponding position in the oligosilicate is empty.

Joining the two open ends of the oligomer in K_0.72_Na_1.71_Ca_5.79_Si_6_O_19_ results in the formation of a closed highly puckered [Si_6_O_18_] ring located in the centre of the aforementioned pseudo-cubic module. This specific structural feature is actually realized in a large number of inorganic compounds belonging to the so-called lovozerite-type of structures (Pekov *et al.*, 2009 ▸). Combeite, which is ideally Na_4_Ca_4_[Si_6_O_18_] is a chemically related member of this group (Fischer & Tillmanns, 1987 ▸). Fig. 3 ▸(*c*) illustrates the corresponding basic module, highlighting its similarity to the present compound.

The computational results indicate that the vibrational spectrum of the measured system can be adequately represented by the idealized fully K-occupied oligosilicate structure. The observed differences in intensity can be explained by the idealized occupation of partially occupied sites, as well as by the 0 K conditions associated with the use of energy-minimized structures. During the measurements, other effects such as luminescence may occur, which could cause slight deviations between the experimental and calculated spectra. The comparison of the molecular electronegativities of the Na- and K-saturated oligo- and cyclo­silicates were compared, and it was found that the latter corresponds to the more stable structure. The oligosilicate exhibits lower stability due to its distinct charge transfer properties in the EEM calculation, which can be directly attributed to the oxygen atoms of the open [Si_6_O_19_] moieties. This is particularly evident in the molecular electronegativity difference considering the number of elements per nm^3^, where the oxygen atoms have the greatest impact on the calculation results. The EEM data indicate that the energy-minimized geometries in this case do not significantly differ from those of the experimental structures using idealized occupancies. Therefore, the outlined EEM implementation offers an efficient framework for analysing the stability of various systems. This study shows that the EEM approach, which is simple and cost-effective, can be extended to the characterization of the quaternary system K_2_O–Na_2_O–CaO–SiO_2_ without pre-optimization of the experimentally determined geometry. The initial assessments can also aid in the preselection of analyses and systems without excessive effort. In the future, our EEM approach could be a used as part of analytical techniques to better understand quaternary systems such as K_2_O–Na_2_O–CaO–SiO_2_.

## 5. Conclusion

Liebau (1985 ▸) used Pauling’s rule of parsimony to explain the decreasing stability of [Si*_m_*O_3*m*+1_] units with increasing *m* in his seminal book on the crystal chemistry of silicates. The rule states that a crystalline compound tends to have the smallest possible number of different constituents or building elements. As the number of chemically non-equivalent [SiO_4_] tetrahedra increases with increasing *m*, a [Si_5_O_16_] unit (*m* = 5) should be less stable than a [Si_2_O_7_] dimer (*m* = 2). It is noteworthy that Liebau (1985 ▸) correlated the (relative) stability of oligomers having a specific value of *m* with the number of the corresponding observed crystal structures. This hypothesis is actually supported when comparing the number of published crystal structures with *m* = 5 and *m* = 2, respectively. Further research could involve EEM calculations to compare the molecular electronegativities of oligomers with different values of *m*. Furthermore, such study could also verify or falsify the existence of a minimum stability point at *m* = 7, as suggested by Liebau (1985 ▸). According to Liebau’s argument, stability is positively correlated with the number of representatives, implying that [Si_6_O_18_] rings should be more stable than [Si_6_O_19_] groups. Our investigation has quantified this assumption using two specific examples.

The new oligosilicate structure type was determined from a single-crystal with a particular chemical composition. However, numerous examples from the mineral kingdom and from synthetic silicates demonstrate extensive cation substitutions between Na and K, and Na and Ca, respectively. It is therefore more than likely that the compound under investigation belongs to a more complex solid-solution series. A detailed analysis of the compositional space of these mixed crystals was not within the scope of this study. Further research could be conducted to examine the growth of single crystals at the compositions of the hypothetical solid-solution series, in conjunction with structural investigations. This could prove a fruitful study, shedding more light on the compound’s structural response to the replacement of the alkali and alkaline-earth cations.

In particular, the EEM calculations have demonstrated that the newly synthesized oligosilicate is notably less stable than its cyclo­silicate counterpart, regardless of whether Na^+^ or K^+^ are present as counterions. The new shielded EEM approach showed that it is a fast and computational cost-effective method for identifying relative stability differences in silicates. Consequently, this approach has the potential to be extended to other silicate systems in the future.

## Supplementary Material

Crystal structure: contains datablock(s) global, I. DOI: 10.1107/S2052520624007352/dk5130sup1.cif

Structure factors: contains datablock(s) I. DOI: 10.1107/S2052520624007352/dk5130Isup2.hkl

Supplementary Tables and Figures. DOI: 10.1107/S2052520624007352/dk5130sup3.pdf

CCDC reference: 2372966

## Figures and Tables

**Table 1:** Experimental details

Crystal data	
Chemical formula	Ca_5.79_K_0.72_Na_1.71_O_19_Si_6_
*M*_r_	771.93
Crystal system, space group	Tetragonal, *P*4_1_22
Temperature (K)	296
*a*, *c* (Å)	7.3659 (2), 32.2318 (18)
*V* (Å^3^)	1748.78 (12)
*Z*	4
Radiation type	Mo *K*α
μ (mm^−1^)	2.49
Crystal size (mm)	0.15 × 0.09 × 0.09

Data collection	
Diffractometer	Xcalibur, Ruby, Gemini ultra
Absorption correction	Analytical (*CrysAlis PRO*, v.1.171.40.84a). Analytical numeric absorption correction using a multifaceted crystal model based on expressions derived by Clark & Reid (1995 ▸).
*T*_min_, *T*_max_	0.767, 0.84
No. of measured, independent and observed [*I* > 2σ(*I*)] reflections	27647, 1788, 1690
*R*_int_	0.055
(sin θ/λ)_max_ (Å^−1^)	0.625

Refinement	
*R*[*F*^2^ > 2σ(*F*^2^)], *wR*(*F*^2^), *S*	0.024, 0.059, 1.11
No. of reflections	1788
No. of parameters	164
Δρ_max_, Δρ_min_ (e Å^−3^)	0.26, −0.28
Absolute structure	Flack (1983 ▸)
Absolute structure parameter	−0.03 (5)

Computer programs: *CrysAlis PRO* (Rigaku Oxford Diffraction, 2020 ▸), *SHELXL97* (Sheldrick, 2008 ▸).

**Table 2:** Atomic coordinates (×10^4^) and equivalent isotropic displacement parameters (Å^2^ × 10^3^) for K_0.72_Na_1.71_Ca_5.79_Si_6_O_19_ *U*_eq_ is defined as one third of the trace of the orthogonalized *U*_*ij*_ tensor. BVS: bond valence sums.

	Wyckoff site	*x*	*y*	*z*	*U*_eq_	BVS (v.u.)	Site populations for the Na, Ca and K ions (%)
M1	8*d*	2656 (1)	7514 (1)	9352 (1)	10 (1)	2.092	96.0 (8) Ca / 4.0 (8) Na
M2	4*c*	147 (1)	147 (1)	8750	9 (1)	2.179	96.4 (9) Ca / 3.6 (9) Na
M3	4*a*	0	30 (1)	0	10 (1)	1.997	93.9 (9) Ca / 6.1 (9) Na
M4	4*a*	0	4637 (1)	0	11 (1)	1.742	95.9 (9) Ca / 4.1 (9) Na
M5	8*d*	9840 (1)	5402 (2)	8781 (1)	14 (1)	1.135	25.2 (7) Ca / 74.8 (7) Na
M6	4*b*	5000	375 (2)	0	17 (1)	1.346	50 Ca / 50 K
M7	4*b*	5000	5004 (7)	0	20 (2)	1.214	21.6 (4) K
Si1	8*d*	2811 (1)	2652 (1)	9256 (1)	8 (1)	4.124	
Si2	8*d*	7162 (1)	3021 (1)	9317 (1)	9 (1)	4.133	
Si3	8*d*	7528 (1)	7359 (1)	9464 (1)	8 (1)	4.083	
O1	8*d*	1835 (3)	4472 (3)	9403 (1)	18 (1)	1.963	
O2	8*d*	9324 (3)	8070 (3)	9232 (1)	15 (1)	1.977	
O3	8*d*	7907 (3)	7488 (3)	9958 (1)	18 (1)	2.018	
O4	8*d*	8108 (3)	2182 (3)	9717 (1)	17 (1)	1.902	
O5	8*d*	7869 (3)	2338 (3)	8873 (1)	20 (1)	1.957	
O6	8*d*	5644 (3)	8268 (4)	9335 (1)	24 (1)	2.002	
O7	8*d*	2032 (3)	745 (3)	9408 (1)	12 (1)	1.899	
O8	8*d*	4975 (3)	2672 (4)	9352 (1)	26 (1)	2.254	
O9	4*c*	2562 (3)	2562 (3)	8750 (1)	29 (1)	2.248	
O10	8*d*	7512 (4)	5192 (3)	9338 (1)	28 (1)	2.253	

**Table 3:** Anisotropic displacement parameters (Å^2^ × 10^3^) for K_0.72_Na_1.72_Ca_5.79_Si_6_O_19_ The anisotropic displacement factor exponent takes the form: −2π^2^[*h*^2^a*^2^*U*_11_ + … + 2hka*b*U_12_].

	*U*_11_	*U*_22_	*U*_33_	*U*_23_	*U*_13_	*U*_12_
M1	11 (1)	10 (1)	9 (1)	0 (1)	1 (1)	−1 (1)
M2	8 (1)	8 (1)	10 (1)	1 (1)	−1 (1)	−1 (1)
M3	7 (1)	8 (1)	16 (1)	0	1 (1)	0
M4	11 (1)	11 (1)	10 (1)	0	3 (1)	0
M5	16 (1)	16 (1)	12 (1)	−2 (1)	−1 (1)	−2 (1)
M6	21 (1)	19 (1)	12 (1)	0	2 (1)	0
M7	18 (3)	25 (3)	17 (2)	0	5 (2)	0
Si1	7 (1)	8 (1)	7 (1)	−1 (1)	0 (1)	−1 (1)
Si2	9 (1)	8 (1)	9 (1)	−1 (1)	0 (1)	−1 (1)
Si3	8 (1)	7 (1)	9 (1)	0 (1)	−1 (1)	−1 (1)
O1	23 (1)	9 (1)	22 (1)	1 (1)	8 (1)	0 (1)
O2	12 (1)	15 (1)	17 (1)	4 (1)	2 (1)	−1 (1)
O3	19 (1)	24 (1)	11 (1)	1 (1)	−1 (1)	−6 (1)
O4	15 (1)	24 (1)	11 (1)	−1 (1)	−1 (1)	6 (1)
O5	30 (1)	22 (1)	9 (1)	−1 (1)	−1 (1)	12 (1)
O6	11 (1)	28 (2)	33 (1)	14 (1)	−2 (1)	0 (1)
O7	14 (1)	9 (1)	14 (1)	−1 (1)	2 (1)	0 (1)
O8	10 (1)	26 (2)	43 (1)	9 (1)	−6 (1)	−3 (1)
O9	41 (1)	41 (1)	7 (1)	−4 (1)	4 (1)	−18 (2)
O10	39 (2)	11 (1)	32 (1)	−7 (1)	11 (1)	−10 (1)

**Table 4:** Selected bond lengths (Å) for K_0.72_Na_1.71_Ca_5.79_Si_6_O_19_ QE: Quadratic elongation; AV: angle variance.

M1—O3	2.264 (2)	M1—O6	2.271 (2)
M1—O1	2.326 (2)	M1—O5	2.360 (2)
M1—O7	2.430 (2)	M1—O2	2.518 (2)
〈M1—O〉	2.362		
			
M2—O2	2.262 (2)	M2—O2	2.262 (2)
M2—O5	2.362 (2)	M2—O5	2.362 (2)
M2—O9	2.516 (3)	M2—O7	2.572 (2)
M2—O7	2.572 (2)		
〈M2—O〉	2.415		
			
M3—O4	2.299 (2)	M3—O4	2.299 (2)
M3—O3	2.429 (2)	M3—O3	2.423 (2)
M3—O7	2.483 (2)	M3—O7	2.483 (2)
M3—O2	2.910 (2)	M3—O2	2.910 (2)
〈M3—O〉	2.530		
			
M4—O1	2.356 (2)	M4—O1	2.356 (2)
M4—O4	2.459 (2)	M4—O4	2.459 (2)
M4—O3	2.609 (2)	M4—O3	2.609 (2)
M4—O10	2.842 (3)	M4—O10	2.842 (3)
〈M4—O〉	2.567		
			
M5—O6	2.306 (2)	M5—O2	2.473 (2)
M5—O10	2.488 (3)	M5—O1	2.577 (3)
M5—O5	2.634 (3)	M5—O5	2.700 (3)
M5—O9	2.8993 (11)	M5—O8	2.935 (3)
〈M5—O〉	2.627		
			
M6—O6	2.687 (2)	M6—O6	2.687 (2)
M6—O8	2.687 (3)	M6—O8	2.687 (3)
M6—O4	2.801 (2)	M6—O4	2.801 (2)
M6—O7	2.916 (2)	M6—O7	2.916 (2)
M6—O3	3.021 (2)	M6—O3	3.021 (2)
〈M6—O〉	2.822		
			
M7—O8	2.703 (4)	M7—O8	2.703 (4)
M7—O3	2.820 (4)	M7—O10	2.820 (4)
M7—O10	2.827 (2)	M7—O10	2.827 (2)
M7—O1	3.049 (3)	M7—O1	3.049 (3)
〈M7—O〉	2.850		
			
Si1—O1	1.593 (2)	Si1—O7	1.594 (2)
Si1—O8	1.624 (2)	Si1—O9	1.6420 (8)
〈Si1—O〉 = 1.614	QE = 1.008	AV = 32.09	
			
Si2—O4	1.590 (2)	Si2—O5	1.603 (2)
Si2—O10	1.621 (2)	Si2—O8	1.635 (2)
〈Si2—O〉 = 1.613	QE = 1.004	AV = 16.24	
			
Si3—O6	1.595 (3)	Si3—O2	1.607 (3)
Si3—O3	1.619 (2)	Si3—O10	1.647 (2)
〈Si3—O〉 = 1.617	QE = 1.006	AV = 27.00	

**Table 5:** EEM parameter sets for the different elements taken from the supplementary material of Joshi *et al.* (2014 ▸) (χ: electronegativity, η: chemical hardness, γ: shielding factor).

Element	χ (eV)	η (eV)	γ (Å^−1^)
Si	4.6988	6.0000	0.8925
O	8.5000	8.3122	1.0898
Ca	−1.9372	6.5275	0.7939
Na	−0.9871	6.7728	0.4000
K	−5.0000	10.4546	0.3343

**Table 6:** Selected bond and torsion angles (°) for K_0.72_Na_1.71_Si_6_O_19_ Si atoms suffixed with ‘a’ are generated from the unlabeled counterpart by application of the twofold rotation axis running through the hexamer.


O1—Si1—O7	119.22 (12)	O1—Si1—O8	112.24 (14)
O7—Si1—O8	107.61 (13)	O1—Si1—O9	106.15 (10)
O7—Si1—O9	103.21 (14)	O8—Si1—O9	107.47 (13)
O4—Si2—O5	117.33 (11)	O4—Si2—O10	106.19 (13)
O5—Si2—O10	107.21 (13)	O4—Si2—O8	108.28 (12)
O5—Si2—O8	109.46 (13)	O10—Si2—O8	107.99 (13)
O6—Si3—O2	117.29 (12)	O6—Si3—O3	112.35 (13)
O2—Si3—O3	107.25 (12)	O6—Si3—O10	109.72 (15)
O2—Si3—O10	101.96 (13)	O3—Si3—O10	107.41 (12)

Si1—O8—Si2	162.72 (17)	Si1—O9—Si1	167.7 (2)
Si2—O10—Si3	165.42 (17)		

Si3—Si2—Si1	100.06 (3)	Si2—Si1—Si1a	95.37 (3)
Si1—Si1a—Si2a	97.37 (3)	Si1a—Si2a—Si3a	100.06 (3)

Si3—Si2—Si1—Si1a	95.68 (3)	Si2—Si1—Si1a—Si2a	−79.96 (3)
Si1—Si1a—Si2a—Si3a	95.68 (3)		

**Table 7:** Coordination sequences {*N_k_*} of the tetrahedrally and octahedrally coordinated nodes as well as the extended point symbols for K_0.72_Na_1.71_Ca_5.79_Si_6_O_19_

	Coordination sequences {*N_k_*}(*k* = 1,…12)												
T or M sites	1	2	3	4	5	6	7	8	9	10	11	12	Extended point symbol
Si1	4	4	16	12	37	26	89	52	149	81	227	115	8.12.8.12.8.16_16_
Si2	4	3	11	9	33	24	75	48	148	77	212	113	8.8.12_5_
Si3	4	4	18	14	41	28	94	53	153	84	232	120	8.8.8.12_2_.12.12
M1	6	6	18	13	47	32	97	53	149	82	238	122	8.8.8.8.12.12.12_2_.12_3_.16_7_. 12.16_6_.16_8_.16_10_.12.16_4_

**Table 8:** Tetragonal unit-cell parameters (from energy minimization at HSESol/pob-DZVP-rev2 level of theory) of the supercell setups used in the EEM calculations

	Oligosilicate		Cyclo­silicate	
Monovalent cation species	Na	K	Na	K
*a* (Å)	37.147	36.948	41.678	42.744
*b* (Å)	37.147	36.948	36.094	37.017
*c* (Å)	32.437	32.752	39.346	40.712
*V* (Å^3^)	44759	44711	59189	64416

## Appendix A Electronegativity equalization method – theoretical overview

Electronegativity (χ) is a chemical property that describes the tendency of charge transfer phenomena between individual atoms. Its counterpart, chemical hardness (η), measures an atom’s ability to resist electron transfer. Electronegativity provides an estimation of the bond formation potential, which is directly related to the structural stability in the ground state (Shankar & Parr, 1985 ▸). Both parameters can be very powerful quantities, providing initial insights into the properties of solid-state structures at low computational cost. It is important to note that these properties can be derived directly from DFT (Argaman & Makov, 2000 ▸; Brink, 2002 ▸; Jones, 2015 ▸; Yu *et al.*, 2016 ▸).

The relationship between electron density ρ and electronegativity χ can be described by the change in ground state energy *E*, as shown in the following equation (1)

Here, *V*(*r*) represents the external potential, *N* is the number of electrons and μ is the chemical potential. According to Shankar & Parr (1985 ▸), the chemical potential is the negative value of electronegativity in equation (2)

The EEM, formulated by Mortier *et al.* (1985 ▸, 1986 ▸) is an efficient framework for computing electronegativity and charge distributions. The basic following formalism is given by equation (3)

where χ_mol_ represents the molecular electronegativity,  and  are the atomic electronegativity and hardness parameters of the *i*th atom, and *q*_*i*_ represents the respective atomic partial charge. To describe the electrostatic interactions for a solid-state system, periodic boundary conditions must be considered, which may be included, for example, by a Madelung contribution (Jones & Templeton, 1956 ▸) obtained via the Ewald summation (Ewald, 1921 ▸; Stenberg & Stenqvist, 2020 ▸). The Coulomb interaction is divided into a short-range contribution carried out in reciprocal space and a long-range contribution in real space. To accelerate the approach, the interaction is simplified by using a Wolf summation (Wolf *et al.*, 1999 ▸) which is well justified via local neutrality. The EEM equations in this formalism are modified by the inclusion of a damping parameter κ and take the form of equation (4):

where *r*_cut_ is a suitable cut-off radius that is set to half of the smallest unit-cell parameter. In recent years, various studies (Demontis *et al.*, 2001 ▸; Zahn *et al.*, 2002 ▸; Fennell & Gezelter, 2006 ▸; Chen *et al.*, 2010 ▸; Gdoutos *et al.*, 2010 ▸; Kannam *et al.*, 2012 ▸; Viveros-Méndez & Gil-Villegas, 2012 ▸; Fukuda & Nakamura, 2012 ▸) have successfully tested this simplified approach. However, finding suitable atomic parameters  and  remains a particular challenge for the EEM approach, especially in non-binary systems like the quaternary system K_2_O–Na_2_O–CaO–SiO_2_.

The ReaxFF reactive force field approach (Senftle *et al.*, 2016 ▸; Leven *et al.*, 2021 ▸) is a especially successful modelling technique for solid-state systems. It is based on charge equilibration schemes, such as the EEM method, and includes a shielding factor γ (Van Duin, 2009 ▸) to dampen overly strong short-range interactions. Parameter sets for χ^0^, η^0^ and γ can be found in the literature for most elements, which have proven adequate for the EEM approach used in this work.

The EEM framework can then be described by equation (5)

where γ_*ij*_ = (γ_*i*_ γ_*j*_)^1/2^ is the geometric mean of the atomic damping parameters taken from the ReaxFF parameterization by Joshi *et al.* (2014 ▸).

## Appendix B Validation of the shielded electronegativity equalization method approach

The EEM approach was validated by comparing the stabilization energies determined for a test set of four different small silica structures containing α-cristobalite (Dera *et al.*, 2011 ▸), α-quartz (Antao *et al.*, 2008 ▸), coesite (Angel *et al.*, 2003 ▸) and stishovite (Hill *et al.*, 1983 ▸) with unit-cell parameters < 15 Å against reference data taken from the literature (Van Genechten *et al.*, 1987 ▸; Van Genechten & Mortier, 1988 ▸; Verstraelen *et al.*, 2012 ▸) (see also Fig. S1). The initial geometries of the aforementioned crystal structures were optimized at DFT level using the *Crystal23* program (Erba *et al.*, 2023 ▸) before conducting the EEM calculations. The HSEsol functional (Schimka *et al.*, 2011 ▸), which is a reliable option for treating solid-state systems, was used in combination with the pob-DZVP-rev2 and pob-TZVP-rev2 basis sets (Vilela Oliveira *et al.*, 2019 ▸). A shrinking factor of 4 was used for Brillouin zone (BZ) sampling, and the convergence criterion for energy was set to 10^−8^ Hartree, which proved to be sufficient. The optimized unit-cell parameters are available in supporting information Table S1 and in Table S2 a comparison between the unoptimized and optimized unit-cell parameters can be found.

The EEM calculations were performed using an in-house developed EEM algorithm capable of treating periodic systems. The EEM framework from equation (5) was applied to the geometry optimized supercell structures. The relevant ReaxFF parameters, taken from Joshi *et al.* (2014 ▸), are listed in Table 5 ▸. The molecular electronegativities of the results were compared to literature values (Van Genechten *et al.*, 1987 ▸; Henry, 1997 ▸), while also assessing the impact of the basis sets applied in the DFT structure optimization.
