# Supplementary material for: K0.72Na1.71Ca5.79Si6O19 – the first oligosilicate based on [Si6O19]-hexamers and its stability compared to cyclo­silicates
Source: Acta Crystallogr B Struct Sci Cryst Eng Mater. 2024 Aug 30;80(Pt 5):474–87. doi: 10.1107/S2052520624007352 (PMC11457099; doi:10.1107/S2052520624007352)
Supplement: Supplementary file 3 [file b-80-00474-sup3.pdf]

STRUCTURAL SCIENCE
CRYSTAL ENGINEERING
MATERIALS

Volume 80 (2024)

Supporting information for article:

$K_{0.72}Na_{1.71}Ca_{5.79}Si_6O_{19}$ – the first oligosilicate based on $[Si_6O_{19}]$-hexamers and its stability compared to cyclosilicates

Volker Kahlenberg, Hannes Krüger, Sonja Garber, Biljana Krüger, Eugen Libowitzky, Stefanie Kröll, Thomas S. Hofer, Josef M. Gallmetzer and Felix R. S. Purtscher

Table S1 Comparison of the optimised unit-cell parameters, Si-O distances and O-Si-O angles for the different SiO_2 polymorphs using different basis sets (DZVP: pob-DZVP-rev2; TZVP: pob-TZVP-rev2).

Optimisation was performed using the program *Crystal23* at the DFT/HSESol level of theory. The last two columns show the minimum and maximum values for the calculated Si-O bond distances and O-Si-O angles, respectively.

	a [Å]	b [Å]	c [Å]	α [°]	β [°]	γ [°]	Si-O [Å]	O-Si-O [°]
α-cristobalite DZVP	4.910	4.910	6.769	90	90	90	1.6312 - 1.6319	107.5 - 112.6
α-cristobalite TZVP	4.966	4.966	6.867	90	90	90	1.6292 - 1.6294	107.9 - 112.5
α-quartz DZVP	4.850	4.850	5.382	90	90	120	1.6300 - 1.6358	107.7 - 111.4
α-quartz TZVP	4.909	4.909	5.417	90	90	120	1.6284 - 1.6329	108.3 - 111.1
coesite DZVP	7.090	12.444	7.220	90	120.73	90	1.6078 - 1.6418	107.6 - 111.3
coesite TZVP	7.155	12.450	7.226	90	120.54	90	1.6103 - 1.6399	107.9 - 110.9

stishovite DZVP	4.166	4.166	2.691	90	90	90	1.7696 - 1.7965	81.0 - 180.0
stishovite TZVP	4.173	4.173	2.689	90	90	90	1.7686 - 1.8019	81.0 - 180.0

Table S2 Comparison of the unit-cell parameters for unoptimised and optimised orthogonal super-cells of the oligo- and the cyclosilicate with different Na/K occupations, along with the corresponding difference between unoptimised and optimised unit-cell parameters.

The number of atoms in the supercells of the oligo- and the cyclosilicate structures are also provided.

**Oligosilicate**	unoptimised	optimised		Δ unopt/opt	
Occupation	*Na/K*	*Na*	*K*	*Na*	*K*
a [Å]	36.830	37.147	36.948	0.317	0.199
b [Å]	36.830	37.147	36.948	0.317	0.199
c [Å]	32.232	32.437	32.752	0.205	0.520
V [Å^3]	43721	44759	44711	1039	990
α [°], β [°], γ[°]	90	90	90	0	0
**Cyclosilicate**	unoptimised	optimised		Δ unopt/opt	
Occupation	*Na/K*	*Na*	*K*	*Na*	*K*

a [Å³]	41.856	41.678	42.744	0.178	0.888
b [Å³]	36.248	36.094	37.017	0.154	0.769
c [Å³]	39.504	39.346	40.712	0.158	1.208
V [Å³]	59935	5 189	64416	746	4481
α [°], β [°], γ[°]	90	90	9ß	0	0
**Number of at-oms**	*O*	*Si*	*Na/K*	*Ca*	*sum*
**Oligosilicate**	1900	601	201	701	3400
**Cyclosilicate**	2592	577	865	577	4608

Table S3 Comparison of observed and calculated Raman bands. The Table gives information about the differences in band positions, the mode symmetry, and mode type (stretching or bending).

Vibrational analysis was conducted using the program *Crystal23* at the DFT/HSESol / pob-DZVP-rev2 level of theory. The vibrational modes at the Brillouin centre of the tetragonal unit cell (point group 422 or D_4) are characterised by their irreducible representation: Γ_{vib} = 48 A_1 + 54 A_2 + 50 B_1 + 52 B_2 + 2*102 E. The Raman-active modes are A_1, B_1 and, B_2, while the IR-active modes are A_2. The E modes are both IR and Raman-active.

Obs [cm⁻¹]	Calc [cm⁻¹]	Difference[cm⁻¹]	Mode Symmetry	Mode Type: stretching(S), bending (B)
109	120	-11	A1	(B) O4-K1-Si2; O4-K1-O6
365	231	134	A1	(B) Ca5-Si3-O6; Ca2-Ca1-K2;<br>Ca1-Ca2-Ca1; Ca3-Ca2-O2
408	315	93	A1	(B) O1-Ca4-Si3<br>(S) Ca5-O1; Ca3-O4-Si2; O4-Ca3-O3; Ca4-Ca3

458	468	-10	A1	(B) Si3-O6-Ca5
537	531	6	A1	(B) O1-Si1-O7; Si2-O10-Ca4; O10-Si3-Ca4; O10-Si2-Ca5;O10-Si3-O6
590	612	-22	A1	(B) Si1-O1-Ca5; O7-Si1-Ca2
649	699	-50	A1	(S) O5-Si2-O4
875	885	-10	A1	(S) Si2-O4
916	915	1	A1	(S) Si1-O1
964	926	38	A1	(S) Si3-O10-Si2
1016	1009	7	A1	(S) Si1-O1; Si1-07
1554	-	-	-	-

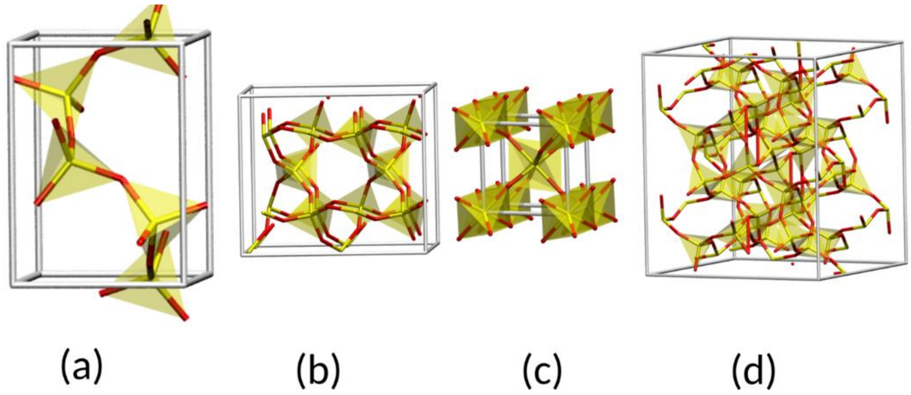

Figure S1 Validation test set of simple silica structures. (a) α-cristobalite, (b) α-quartz, (c) coesite, (d) stishovite.

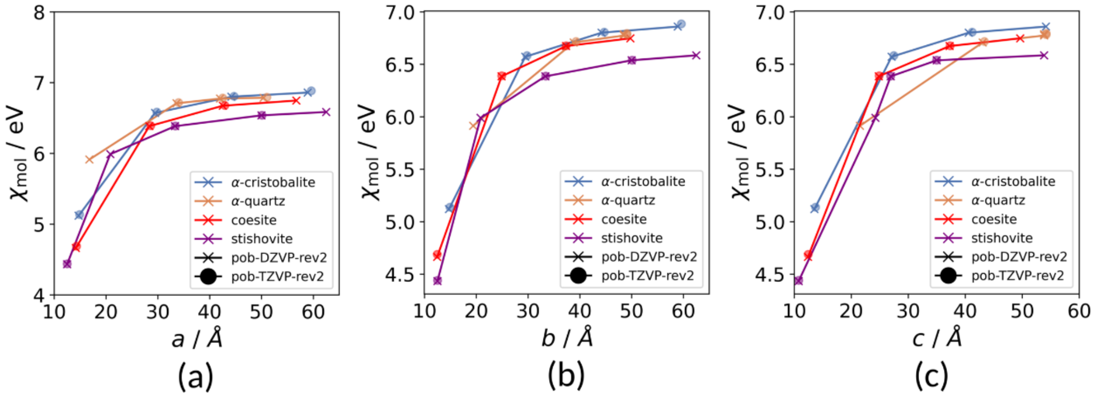

Figure S2 Comparison of molecular electronegativities with supercell unit-cell parameters *a*, *b* and *c* for various silica structures used for validation. Additionally, a comparison between the pob-DZVP-rev2 and pob-TZVP-rev2 basis sets is presented.

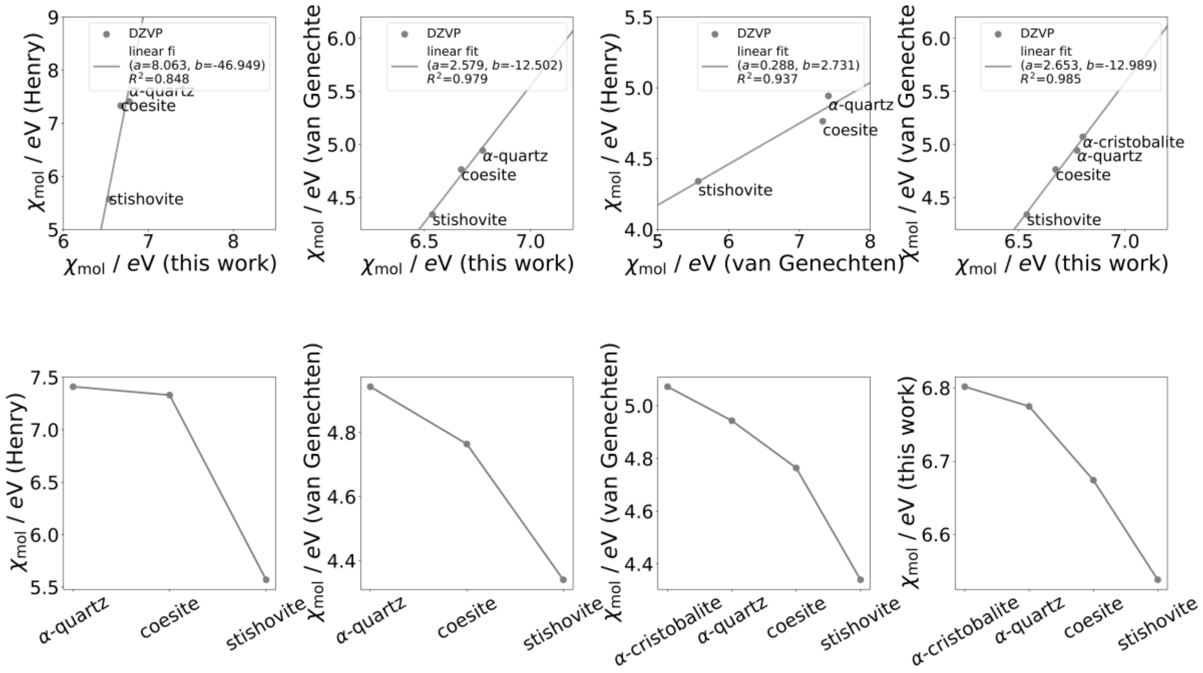

Figure S3 Comparison of molecular electronegativities computed using our EEM approach with literature values from Henry (1997) and Van Genechten *et al.* (1987).
